# An integrated epigenomic-transcriptomic landscape of lung cancer reveals novel methylation driver genes of diagnostic and therapeutic relevance

**DOI:** 10.7150/thno.58385

**Published:** 2021-03-11

**Authors:** Xiwei Sun, Jiani Yi, Juze Yang, Yi Han, Xinyi Qian, Yi Liu, Jia Li, Bingjian Lu, Jisong Zhang, Xiaoqing Pan, Yong Liu, Mingyu Liang, Enguo Chen, Pengyuan Liu, Yan Lu

**Affiliations:** 1 Sir Run Run Shaw Hospital and Institute of Translational Medicine, Zhejiang University School of Medicine, Hangzhou, Zhejiang 310016, China.; 2 Center for Uterine Cancer Diagnosis & Therapy Research of Zhejiang Province, Women's Reproductive Health Key Laboratory of Zhejiang Province, Department of Gynecologic Oncology, Women's Hospital and Institute of Translational Medicine, Zhejiang University School of Medicine, Hangzhou, Zhejiang 310006, China.; 3 Department of Mathematics, Zhejiang University, Hangzhou, Zhejiang 310027, China.; 4 Center of Systems Molecular Medicine, Department of Physiology, Medical College of Wisconsin, Milwaukee, WI 53226, USA.; 5 Cancer center, Zhejiang University, Hangzhou, Zhejiang 310013, China.

**Keywords:** DNA methylation, driver genes, epigenomics, lncRNA, lung cancer, miRNA, reduced representation bisulfite sequencing, transcriptomics, transcription factor

## Abstract

**Background:** Aberrant DNA methylation occurs commonly during carcinogenesis and is of clinical value in human cancers. However, knowledge of the impact of DNA methylation changes on lung carcinogenesis and progression remains limited.

**Methods:** Genome-wide DNA methylation profiles were surveyed in 18 pairs of tumors and adjacent normal tissues from non-small cell lung cancer (NSCLC) patients using Reduced Representation Bisulfite Sequencing (RRBS). An integrated epigenomic-transcriptomic landscape of lung cancer was depicted using the multi-omics data integration method.

**Results:** We discovered a large number of hypermethylation events pre-marked by poised promoter in embryonic stem cells, being a hallmark of lung cancer. These hypermethylation events showed a high conservation across cancer types. Eight novel driver genes with aberrant methylation (e.g., PCDH17 and IRX1) were identified by integrated analysis of DNA methylome and transcriptome data. Methylation level of the eight genes measured by pyrosequencing can distinguish NSCLC patients from lung tissues with high sensitivity and specificity in an independent cohort. Their tumor-suppressive roles were further experimentally validated in lung cancer cells, which depend on promoter hypermethylation. Similarly, 13 methylation-driven ncRNAs (including 8 lncRNAs and 5 miRNAs) were identified, some of which were co-regulated with their host genes by the same promoter hypermethylation. Finally, by analyzing the transcription factor (TF) binding motifs, we uncovered sets of TFs driving the expression of epigenetically regulated genes and highlighted the epigenetic regulation of gene expression of TCF21 through DNA methylation of EGR1 binding motifs.

**Conclusions:** We discovered several novel methylation driver genes of diagnostic and therapeutic relevance in lung cancer. Our findings revealed that DNA methylation in TF binding motifs regulates target gene expression by affecting the binding ability of TFs. Our study also provides a valuable epigenetic resource for identifying DNA methylation-based diagnostic biomarkers, developing cancer drugs for epigenetic therapy and studying cancer pathogenesis.

## Introduction

Lung cancer is the leading cause of cancer mortality worldwide and non-small cell lung cancers (NSCLCs) account for about 85% of lung cancers 1, 2. Although there are new agents for the treatment of NSCLC patients, the 5-year survival rate is still estimated at 15% 1, 3, 4. To reduce the high lethality of NSCLCs, many significant efforts have been made. Previous studies have reported causal genetic alterations, from somatic point mutations to large structure variations, involved in the carcinogenesis of NSCLCs 5-9. Based on these findings, various therapeutic strategies have been developed for NSCLC treatment, such as combination cisplatin-based chemotherapy with the anti-angiogenic bevacizumab 10, 11, the use of tyrosine kinase inhibitors to treat EGFR-mutated, ALK or ROS1-rearranged NSCLC patients 12-15. However, owing to the primary, adaptive and acquired resistance of these drugs, the target treatment is not effective for all NSCLC patients 16. DNA methylation alterations commonly occur in cancer and are involved in tumor initiation and progression. Aberrant promoter CpG methylation provides a selective mechanism for the regulation of tumor suppressor genes and oncogenes in cancer instead of genetic mutations 17. However, these methylation changes could be inherently reversed by agents, in contrast with genetic mutations 18. Thus, identification of epigenetically modulated genes opens new avenues for epigenetic therapies and for discovery of novel drug targets.

In NSCLC, CDKN2A was the first reported gene whose downregulated expression in lung carcinogenesis was predominantly attributed to promoter hypermethylation 19. Afterwards, several studies described several other epigenetically regulated genes, such as FHIT, DAPK and RASSF1A 20-26. However, these earlier studies only investigated a single gene or a small list of genes. The advent of high throughput next-generation sequencing (NGS) for analyzing DNA methylome and transcriptome has offered the unique ability to analyze methylation changes and detect methylation driver events at a genome-wide scale 27-29.

In this study, we analyzed genome-wide methylation profiles of ~ 2 million CpG sites spanning more than 19,600 genes and noncoding regions using Reduced Representation Bisulfite Sequencing (RRBS) technology 30. RRBS combines restriction enzymes and bisulfite sequencing to enrich for DNA segments with a high CpG content and regulatory potentials. It is an efficient and cost-effective technique for analyzing the genome-wide methylation profiles on a single nucleotide level. Using this technique and the multi-omics data integration method, we pinpointed several novel methylation driver protein coding genes and noncoding RNAs that could be potential targets for epigenetic therapy. Furthermore, we detected sets of transcription factor (TF) binding motifs located in differentially methylated regions (DMRs) which regulated target gene expression by affecting the binding ability of TFs in lung cancer.

## Materials and Methods

### Reduced representation bisulfite sequencing (RRBS)

The study was approved by the institutional review board of Sir Run Run Shaw Hospital at Zhejiang University (Hangzhou, China). Eighteen lung tumor tissues and adjacent normal tissues were collected from NSCLC patients (**Table S1**). All samples used in the current study were obtained at the time of diagnosis before any treatment was administered. Genomic DNA of these tissues was extracted and then treated according to the RRBS library preparation protocols as we previously described 30 with modifications to allow multiplexing 31. Paired-end sequencing with 100bp was performed on the Illumina HiSeq 2000 according to manufacturer's protocol.

### Bioinformatics analyses of RRBS data

Bisulfite sequencing reads were pre-processed with Trim Galore (http://www.bioinformatics.babraham.ac.uk/projects/trim_galore/). Both adapters and sequences with low quality (base quality < 20) were removed before the analysis. The trimmed reads were then aligned to the human reference genome (hg19) and the methylation status of each CpG was determined using Bismark (v0.14.1) with default parameters 32 (**Table S2**). The unconverted cytosines at fill-in 3′ MspI sites of sequencing reads were used to estimate the bisulfite conversion rate. For each CpG site with at least 5×coverage, the methylation rate, C/(C+T), was calculated. We merged the 18 tumor-normal sample pairs based on CpG coordinates, yielding 2,574,098 CpG sites that covered at least ten paired samples. CpG sites in the heterosome and CpG sites overlapped with SNP (dbSNP build 142) were filtered, and 2,166,853 were retained for subsequent analysis. The remaining missing methylation values were imputed using k-nearest neighbors in the CpGs space (http://bioconductor.org/packages/release/bioc/html/impute.html).

Next, we used metilene (Version 0.23) 33 to identify DMRs between tumor and matched noncancerous tissues. These DMRs were further examined for significance using the Wilcoxon rank sum test for a paired dataset. The final DMRs were determined using the following threshold: at least ten CpGs in the DMRs, at least 10% differences in methylation, and false discovery rate (FDR) < 0.05. We performed an unsupervised hierarchical cluster analysis on the CpG sites' methylation using ward linkage and Euclidean distance. The Metascape software (http://metascape.org) was used to conduct Gene Ontology (GO) analyses according to the standard protocol.

### RNA-seq analysis

mRNA sequencing libraries were prepared for the same set of lung tumor and adjacent normal tissues using TruSeq RNA Sample Preparation Kit from Illumina as we described previously with modifications 31 (**Supplementary Materials and Methods**). Transcriptome data were mapped with Tophat v2, using the spliced mapping algorithm 34. A set of both known and novel transcripts was constructed and identified using Cufflinks 35. Reads per kilobase of exon per million fragments mapped (RPKM) was used to quantify gene expression level. Finally, differentially expressed genes were obtained by using paired t-test with FDR < 0.05.

### Identification of lncRNAs

Identification of long noncoding RNAs (lncRNAs) followed with the workflow described previously with modifications 36. In brief, we first filtered transcripts with single exon or length <160 bp. The protein coding potential of the remaining transcripts was evaluated by PhyloCSF according to the genome alignments of chimp, rhesus, mouse, guinea, pig, cow, horse and dog 37. Transcripts with PhyloCSF scores greater than 50 were removed for their high coding potential. Moreover, we discarded transcripts with complete branch lengths (CBL) >0 and open reading frames (ORF) of >150 amino acids. Transcripts with CBL scores equal to 0 because of low sequence alignments were also removed if they contained an ORF with more than 50 amino acids. Finally, we employed blastx with repeats masked to analyze the remaining transcripts and removed those with a median of the E-value <1e-18. The identified transcripts were classified into different categories according to the HUGO Gene Nomenclature Committee.

### Small RNA sequencing analysis

Small RNA sequencing libraries were prepared with the TruSeq Small RNA Sample Preparation Kit from Illumina as we described previously with modifications 38 (**Supplementary Materials and Methods**). The raw short reads were trimmed by Trim Galore as described above. The trimmed reads were aligned to the human reference genome (NCBI build 37) using the Burrows-Wheeler Aligner 39. Only one nucleotide mismatch was allowed in the mapping process. Read counts for miRNAs (miRBase v21) were extracted from alignment files using BEDtools (http://bedtools.readthedocs.io/en/latest). miRNA expression levels were quantified by Reads Per Million (RPM) mapped reads and then normalized with log2(RPM+1).

### Annotation with genomic features

To gain a better insight into the genomic features of differential methylation, the identified DMR sets were intersected with a collection of functional regions, including the 15 chromatin states in embryonic stem cells (ESCs), lung-related tissues and cells established by the NIH Roadmap Epigenetics Mapping Consortium (http://egg2.wustl.edu/roadmap/web_portal/). The genomic coordinates of CpG islands (CGIs) based on GRCh37 were downloaded from the UCSC Genome Browser. The protein-coding gene and lncRNA annotation were obtained from GENCODE (v28, GRCh37). The genomic annotation of primary miRNAs were downloaded from miRBase 21 40. The promoters were defined as ±2kb of transcription start site (TSS) of genes and noncoding RNAs (ncRNAs). The feature was annotated to the DMR if it was overlapped by at least 50% bases of that DMR. We further defined ESC-marked chromatin states of DMR, which represent that states were recurrently annotated to the DMR in at least half of our available ESCs (n=3). The chromatin landscape of the selected genes was visualized using the WashU EpiGenome Browser tool (http://epigenomegateway.wustl.edu/browser/).

### Enrichment analysis

The fold enrichment of overlap for a variety of functional annotations (e.g., CGI and given states) over the set of DMRs was calculated using a previously described method 6; briefly, as the odds ratio of the joint probability of overlapped regions between the DMR set and a given state and the product of marginal probability of the DMR set and the marginal probability of the state in the whole genome. The fold enrichment is estimated as follows:


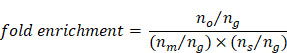


where 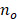 indicates the number of overlapped bases between the DMR set and the state, 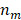 represents the number of bases in the DMR set, and 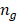 is the number of bases in the genome.

In order to make comparisons across different states, we normalized the enrichments across all 15 states and used a normalized enrichment score (E) for a given state (*i*):


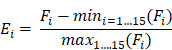


where 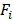 is the fold enrichment for a given state. The normalized enrichment scores ranged from 0 to 1.

### Data collection and analysis of HM450 methylation and RNA-seq data from the TCGA cohorts and CCLE database

The HM450 DNA methylation data of 21 cancer types in The Cancer Genome Atlas (TCGA) were downloaded from the ICGC Data Portal (https://dcc.icgc.org) (**Table S3**). Beta values ranging from 0 to 1 were used to measure DNA methylation levels. The values close to 0 mean a low level of methylation and 1 represents high-level methylation. CpG sites in the heterosome and overlapped with SNP (dbSNP build 142) were removed. In addition, CpG sites with more than 10% missing values across all samples were discarded. The remaining missing methylation beta values were estimated using k-nearest neighbors in the CpGs space (http://bioconductor.org/packages/release/bioc/html/impute.html) and then used for subsequent analysis.

The TCGA mRNA expression data of 21 cancer types were downloaded from the ICGC Data Portal. Then the expression values of protein-coding genes were normalized using RPKM. Low-expressed protein-coding genes with 90th quantile RPKM < 1 were removed and the remaining log2-transformed RPKM values were used for subsequent analysis.

HM450 DNA methylation data of 1,028 cancer cell lines were downloaded from the COSMIC database and RNA-seq data of 781 cancer cell lines were from the Encyclopedia of DNA Elements (ENCODE) and Cancer Cell Line Encyclopedia (CCLE) databases (E-MTAB-2770). In total, 455 cancer cells have both HM450 methylation and RNA-seq data.

These HM450 probes were then mapped to functional genomic regions based on their genomic coordinates in GRCh37. In this way, we defined: (1) DMR probes, located within the DMRs; and (2) gene probes, located within gene promoter regions (+/- 2kb from TSS).

### Identification of methylation driver sets

We used a discovery-validation strategy to pinpoint high-confidence methylation driver genes. The discovery-stage analyses were performed on our RRBS data and matched RNA-seq data. Specifically, we first identified gene and DMR pairs in which the DMR was overlapped with the gene's promoter. Then, we filtered those gene-DMR pairs where the genes were not differentially expressed in tumors compared with normal tissues (FDR < 0.05 and fold change > 2). Finally, we calculated the Spearman correlation coefficients (rho) between methylation changes and gene expression for each of the remaining gene-DMR pairs. For multiple DMRs for each promoter, we chose one DMR that had the most negative association with matched gene expression to capture the most functionally relevant events (epigenetic silencing). In the discovery stage, we obtained a primary gene list, which consisted of 190 protein coding genes with rho values < -0.3 and FDR < 0.05.

Next, for the validation-stage analyses on TCGA Human450methylation (HM450) microarray and RNA-seq data from two NSCLC subtypes (lung adenocarcinoma, LUAD; and lung squamous cell carcinomas, LUSC), we found that the promoter of 133 out of the primary gene set was covered by at least one HM450 CpG probe and these gene-probe pairs were used for subsequent analysis. Similarly, the Spearman correlations between CpG probe methylation and gene expression across all samples were calculated for these gene-probes pairs. If multiple CpGs were annotated to one gene promoter, the CpG probe with the most negative correlation was selected. Finally, we screened out a secondary gene set of 81 genes with rho values < -0.3 and FDR < 0.05 in at least one cancer type (LUAD or LUSC). Among them, 31 genes were identified in both LUAD and LUSC, including 20 hypermethylated ones and 11 hypomethylated ones. Hypermethylation in a promoter was a classical paradigm to induce gene silence, thus the list of 20 genes was chosen as our final high-confidence methylation drivers.

Similarly, we also identified 13 methylation driver ncRNAs (eight lncRNAs and five miRNAs) in lung cancer using the strategy as described above.

### Analysis of regulatory transcription factors

Genomic coordinates of binding motifs of 84 TF groups were obtained from (http://compbio.mit.edu/encode-motifs/). The 190 DMRs in promoters of genes inversely correlated with gene expression were a set of regions of interest. A hypergeometric test was employed to evaluate whether a particular TF binding motif was more enriched in the set of regions of interest than other TF binding motifs. All the identified DMRs between tumor and normal samples served as a background for this test.

In order to identify regulatory TFs, the Spearman rank correlation test was used to calculate the correlation between the expression of TFs corresponding to enriched motifs and the expression of targeted genes (FDR < 0.05).

Activity score 41 was defined as y = ρ×log2(fold change), where ρ was the Spearman correlation coefficient between DMR and the targeted gene. The average activity score was calculated for binding motifs of TFs with multiple binding sites in the DMR of targeted gene.

### ROC analysis

ROC analysis was performed using a random forest model in the caret R packages 42. The samples in the TCGA cohorts (LUAD: Tumor 460, Normal 32; LUSC: Tumor 371, Normal 41) were randomly divided into equal-size training and testing sets. Based on the model parameters obtained from the training sets, the testing sets and an independent lung cancer dataset were used to evaluate the model performance. A ROC plot was conducted using pROC R packages (www.r-project.org).

### Pyrosequencing methylation analysis

Genomic DNA from 23 pairs of lung cancer tissues and matched adjacent normal tissues was isolated using a PureLink Genomic DNA Mini Kit (Invitrogen) according to the manufacturer's protocol. 500ng of DNA underwent bisulfite treatment using the EZ DNA methylation-Gold^TM^ Kit (Zymo Research) according to the manufacturer's protocol to convert all unmethylated cytosine to uracil while leaving 5-methylcytosine unaltered, and was then eluted in 40 μl of DNase-free water. Primer sequences for methylation specific PCR (MSP) and sequencing primers were designed using the Pyromark Assay Design 2.0 Software (Qiagen) (**Table S4**). Bio-primer sequences and sequencing primers were oligo-synthesized (Invitrogen, Beijing, China). DNA methylation level of eight DMRs was analyzed by MSP and pyrosequencing. MSP products were observed at 2% agarose gels before pyrosequencing. Pyrosequencing was done following the instructions of the manufacturer of the pyrosequencing device, PyroMark Q24 (Qiagen).

The methylation values of all the CpG sites within the DMRs were averaged and paired *t*-test was used to infer whether the differences between tumor and normal tissues were statistically significant.

### 5-aza-dC treatment

Lung cancer cell lines A549, Calu1, H1299 and Hop62 were grown in 1640 medium supplemented with 10% FBS and 1% penicillin-streptomycin (Life Technologies) and incubated in 5% CO2 at 37°C. The first day, cell lines were seeded in a 6-well plate (2×10^5^/well) and the second day were treated with 5-aza-2'-deoxycytidine (Sigma) at different final concentrations for 4 days; culture medium was changed every 2 days with new drugs added. Then, cell pellets were harvested for DNA and RNA extraction. Total RNA was extracted using Trizol (Invitrogen) and then the mRNA expression levels of target genes were evaluated using real-time PCR (Takara, RR420A). Genomic DNA was isolated using the PureLink Genomic DNA Mini Kit (Invitrogen, K1820-02) and then pyrosequencing methylation analysis was performed as described above.

### *In vitro* cell-based assays

Detailed information regarding *in vitro* cell-based assays including siRNA knockdown, gene overexpression, cell proliferation and clone formation, and cell migration is provided in **Supplementary Materials and Methods and Table S5**.

## Results

### Genome-wide DNA methylation changes in lung cancers

To investigate aberrations of DNA methylation in primary lung tumors, RRBS was conducted in eighteen tumors and matched adjacent normal lung tissues, including nine lung LUADs and LUSCs (**Table S1**). On average, approximately 22 million reads were uniquely mapped to the reference DNA genome and the bisulfite conversion rate was 0.986 (**Table S2**). 2,574,098 CpG sites that covered at least 10 paired samples were identified. CpG sites in the heterosome and overlapped with a database of single nucleotide polymorphisms (dbSNP build 142) were removed, and the remaining 2,166,853 CpG sites were used for downstream analyses. To assess the reproducibility of RRBS, the genome-wide CpG methylation values between each sample were compared. The average Pearson's correlation coefficient between normal samples was above 0.95 (**Figure S1**), indicating excellent reproducibility of RRBS. Compared with normal samples, the correlation coefficient between tumor samples or between tumor-normal pairs was smaller (**Figure S1**), showing genome-wide DNA methylation changes occurred between tumor-normal pairs, as well as among tumors.

**Figure 1:**
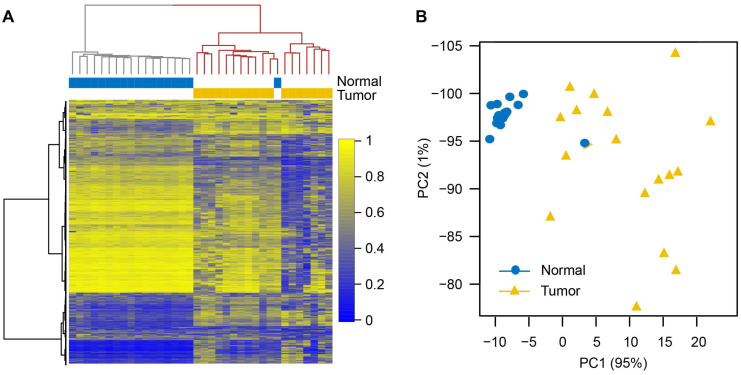
** Comparison of DNA methylation alterations in lung tumor and matched normal tissues.** (**A**) Unsupervised hierarchical clustering based on methylation levels of the top 1% CpG sites (n = 21,668) that varied most across 18 normal/tumor pairs. Columns are samples and rows are CpG sites. (**B**) Principal component analysis of 18 normal/tumor pairs based on methylation levels of all CpG sites (n = 2,166,853).

The top 1% of CpG sites that varied most across all samples were selected to perform unsupervised hierarchical clustering. As expected, the NSCLCs and matched normal samples were clearly segregated, with the exception of one normal sample (**Figure 1A**). Principal component analysis (PCA) using all CpG sites also showed similar segregation between the two groups (**Figure 1B**). These results indicated substantial methylation differences between NSCLCs and normal tissues. We observed that the variation within NSCLCs is much larger than that within normal samples (**Figure 1B**), suggesting intra-tumor DNA methylation heterogeneity in NSCLCs. Moreover, the NSCLCs revealed a lower proportion of highly methylated CpG sites (>80%), and a similar fraction of intermediate methylation (10%-80%) and low methylation (<10%) compared to matched normal ones (**Figure S2A**), showing hypomethylation on a genome-wide scale in NSCLCs. However, enormous methylation gains were observed in NSCLCs at CpG sites within CGIs that had low methylation levels in normal samples (< 10%; **Figure S2B**). Then, we compared DNA methylation differences between lung tumors and normal lung tissues for 15 chromatin states which were defined in ESCs 6. We found that heterochromatin states (Het) had the greatest hypomethylation in NSCLCs compared to their normal counterparts. In contrast, methylation levels of bivalent regulatory states (TssBiv, BivFlnk and EnhBiv) and repressed polycomb states (ReprPC) showed a small increase in NSCLC tumors (**Figure S2C**).

### Cancer-specific hypermethylated regions occurred primarily at genes with poised-promoter in embryonic stem cells

**Figure 2:**
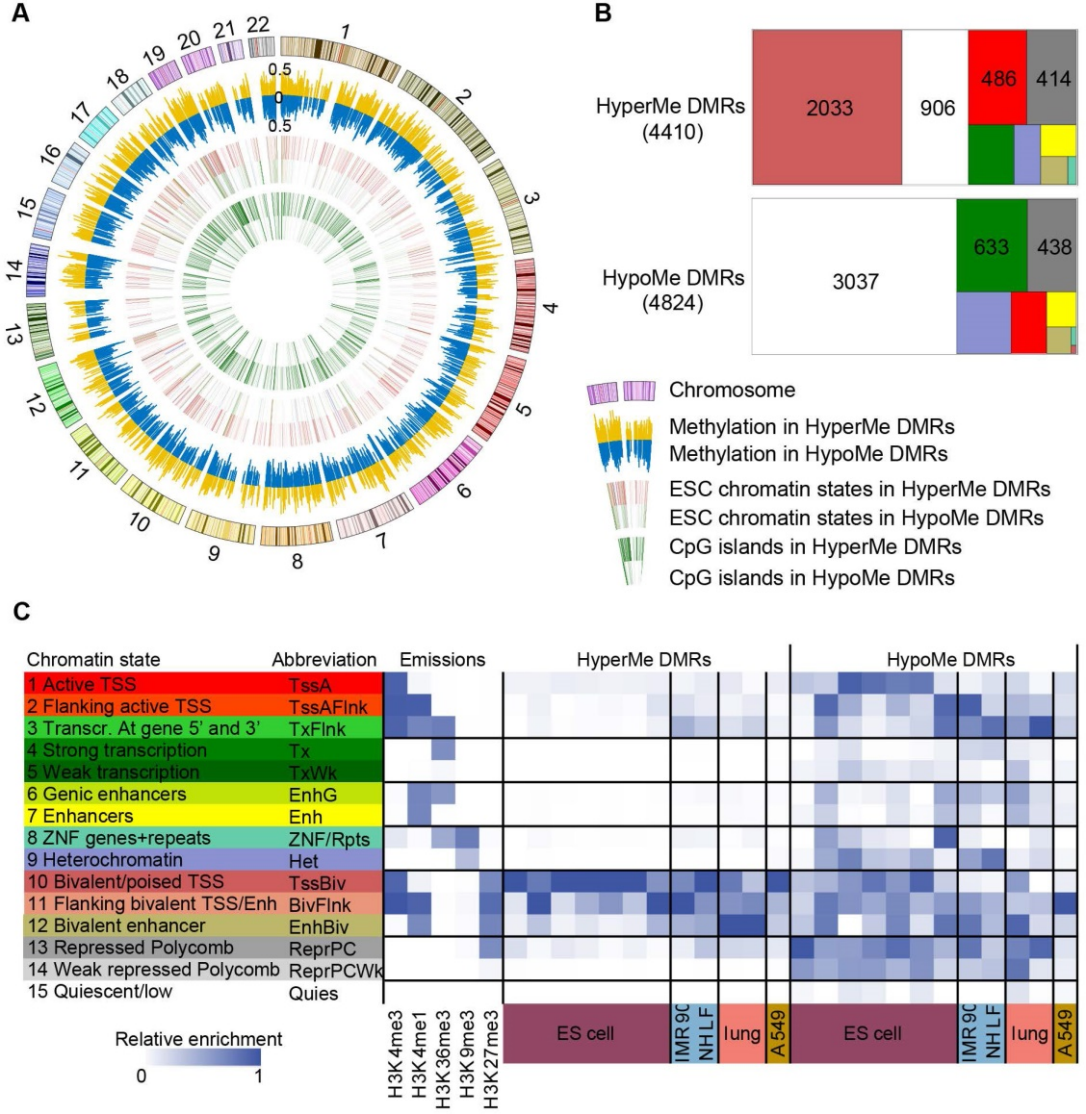
** Characteristics of differentially methylated regions.** (**A**) Circular plot of 4,410 hypermethylated (hyperMe) and 4,824 hypomethylated (hypoMe) differentially methylated regions (DMRs). ESCs, embryonic stem cells. (**B**) Distribution of hyperMe-DMRs and hypoMe-DMRs within the region of 15 chromatin states defined in ESCs. Definitions of 15 chromatin states are shown in Figure 2C. (**C**) Definitions of 15 chromatin states and histone mark probabilities. (**D**) Enrichments of DMRs in ESCs, IMR90, NHLF, lung tissues, and A549 cells across 15 chromatin states.

To identify DMRs in NSCLCs, the whole RRBS dataset was analyzed by an unbiased circular binary segmentation method 43. In total, 4,410 hypermethylated DMRs (hyperMe-DMRs) and 4,824 hypomethylated DMRs (hypoMe-DMRs) were detected in tumors compared to matched normal tissues (**Figure 2A** and **Table S6**). In our study, similar amounts of hypo- and hypermethylation were uncovered in lung cancer, which was in line with previous reports in colon cancer 44. The hyperMe-DMRs were of smaller size (mean values = 215 bp *versus* 263 bp, P = 2.2×10^-16^) and contained more CpG sites (mean values = 22 *versus* 13, P = 2.2×10^-16^) than hypoMe-DMRs (**Figure S3A-B**).

Although both hypermethylation and hypomethylation changes occurred in lung cancer, there were significant differences in the genomic regions and the activity states that were modified. The majority (75.5%) of hyperMe-DMRs were located within CGI, while only 16.1% of hypoMe-DMRs overlapped with CGI (**Figure 2A** and **Table S6**). Moreover, by integrating analysis with 15-chromatin states across ESC epigenomes generated from five histone modification marks, we discovered that hyperMe-DMRs were strongly enriched in poised promoters (TssBiv and BivFlnk) of ESCs, whereas hypoMe-DMRs did not show significant enrichment across the activity states (**Figure 2** and**Table S6**). In lung cancer, 46.1% (2,033) of hyperMe-DMRs were marked by poised promoters in ESCs, which covered promoters of 995 genes including well-known aberrantly methylated homeobox gene cluster 45 (**Figure S4**). Enrichment analysis of Gene Ontology and molecular signature found that these genes were significantly enriched in developmental processes, stem cell differentiation, tri-methylated H3K4 and H3K27, and hypermethylated genes in other cancer types (**Figure S5A-B,** and **Table S7**).These findings demonstrate that genes targeted by a bivalent promoter chromatin pattern (repressive mark H3K27me3 and active mark H3K4me3) in ESCs tend to be de novo methylated in lung cancer.

### HyperMe-DMRs in lung cancer are recurrently hypermethylated in various primary cancers and cancer cell lines

**Figure 3:**
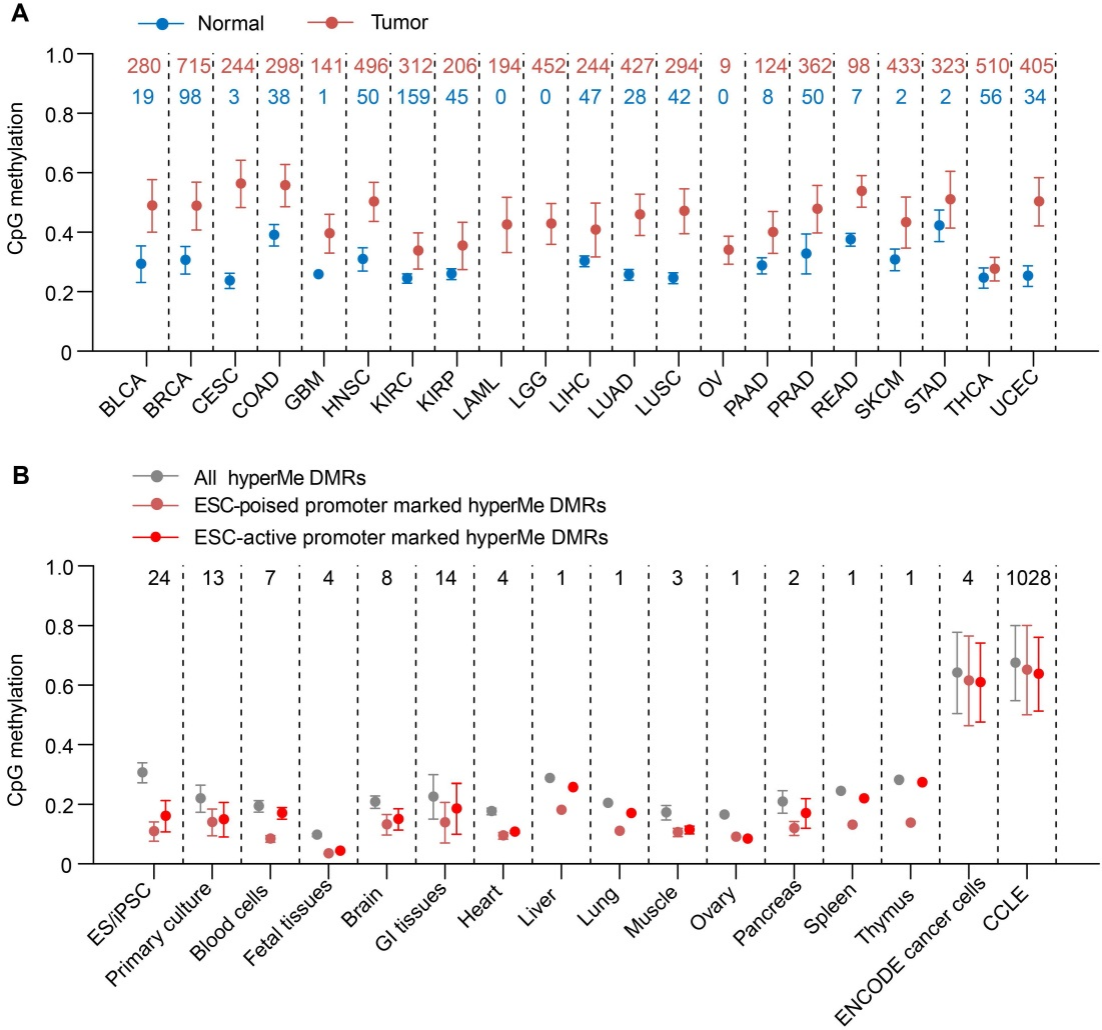
** HyperMe-DMRs are recurrently hypermethylated.** (**A**) Methylation levels of total hyperMe-DMRs between tumor and normal samples across 21 cancer types. Data are shown as mean ± SD. The top digits signified number of tumor (red) and normal (green) samples analyzed. (**B**) Methylation levels of total hyperMe-DMRs and the other set of hyperMe-DMRs (i.e., ESC-poised and ESC-active promoter marked hyperMe-DMRs) across tissues, cell types and cancer cell lines. Data are shown as mean ± SD. The top digits signified number of tissues/cells.

Then we determined if these hyperMe-DMRs in lung cancer were also hypermethylated in other tumor types. By investigating the methylation levels of CpG sites within hyperMe-DMRs from methylation array data of 21 cancer types in TCGA, we observed that the hyperMe-DMRs in our lung cancer data remained hypermethylated in the TCGA lung cancer dataset as well as other primary cancers (**Figure 3A**). Strikingly, we also found that methylation levels of these hyperMe-DMRs in cancer cell lines from the ENCODE project and CCLE database 6, 46, were significantly increased compared to that in ESCs, primary cells from peripheral blood, primary culture cell lines, and primary tissues from the NIH Roadmap Epigenomics Consortium (**Figure 3B**). These results highlight that the hyperMe-DMRs we identified are highly relevant to tumor development and progression. In addition, hyperMe-DMRs targeted by bivalent or active promoters in ESCs also showed hypermethylation in TCGA primary cancers and cancer cell lines (**Figure 3B and Figure S6A-B**). Overall, the above data indicate that a group of development-associated genes, mainly marked by ESC poised promoters, show highly conserved hypermethylation across cancer types and cancer cell lines.

### DNA methylation of ESC-poised promoter marked genes correlates more strongly with gene expression than ESC-active promoter marked genes

**Figure 4:**
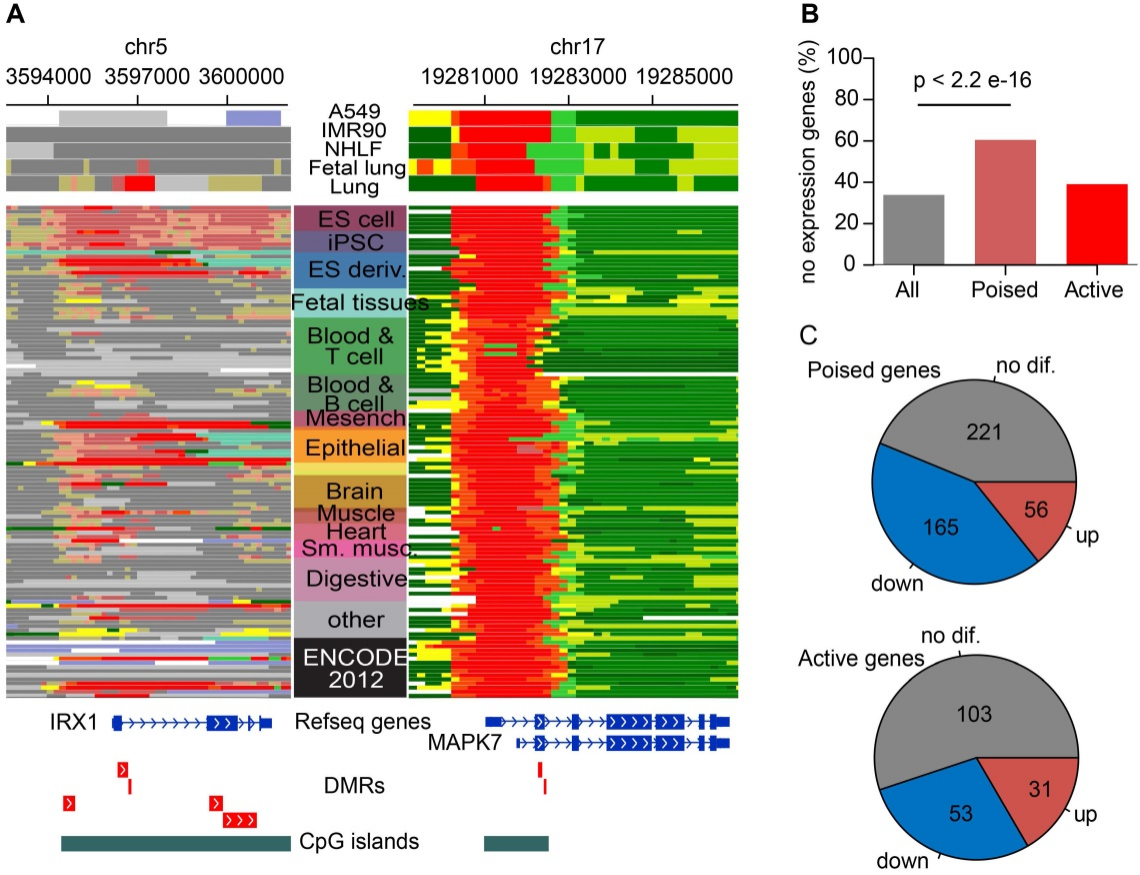
** Functional characterization of hyperMe-DMRs subtypes.** (**A**) Examples of two hyperMe-DMRs subtypes, one is pre-marked by poised promoter (IRX1, poised-hyperMe DMRs) and the other by active promoter (MAPK7, active-hyperMe DMRs) in ESCs. Annotations of chromatin state across 127 reference epigenomes including ESCs and lung-related tissues/cells are shown. (**B**) Fraction of no/bottom-expression genes for all annotated genes and genes harboring poised-hyperMe DMRs and active-hyperMe DMRs in their promoters. (**C**) Distribution of no differential expression, upregulation and downregulation for genes harboring poised-hyperMe DMRs and active-hyperMe DMRs in their promoters.

It was a classical regulatory paradigm that DNA hypermethylation at promoters were generally correlated with gene silencing 47, thus hyperMe-DMRs at promoter regions were our focal point. There were 2,519 (57.1%) hyperMe-DMRs located within promoters, including two types; one where its promoter was marked by poised chromatin states in ESCs (2,033, 46.1%) and another by active chromatin states in ESCs (486, 11%) (**Figure 2B**). Examples included hyperMe-DMRs within IRX1 and MAPK7 promoters (**Figure 4A**), targeted by bivalent and active states, respectively, showing differential chromatin modification in ESCs. Furthermore, we annotated the two types of hyperMe-DMRs by 19,901 protein coding genes (GENCODE V28), and obtained 995 ESC-poised marked genes and 307 ESC-active marked genes, respectively. By integrating analysis with corresponding RNA-seq data, we found that ESC-poised marked genes were significantly enriched in genes with no or low transcript levels in both lung normal and tumor tissues (*P* = 2.2×10^-16^), whereas ESC-active marked genes showed no significant enrichment in those genes (**Figure 4B**). This finding suggests that most ESC-poised marked genes were inactive in normal tissues and generally remain inactive in tumor tissues. This is probably due to switching of the epigenetic silence mechanism where the silenced genes in normal tissues were inactive by de novo methylation in cancer 48-51. For the 393 ESC-poised marked genes and 187 ESC-active marked genes that were expressed in both lung normal and cancer tissues, there were more significantly downregulated genes among the ESC-poised marked genes than the ESC-active marked genes (42% versus 28%, *P* = 0.0018) (**Figure 4C**). Taken together, the results indicated that methylation levels of ESC-poised marked genes are more predictive of their transcript levels over ESC-active marked genes.

### Integrative analysis identified eight novel methylation-silenced genes in lung cancer

**Figure 5:**
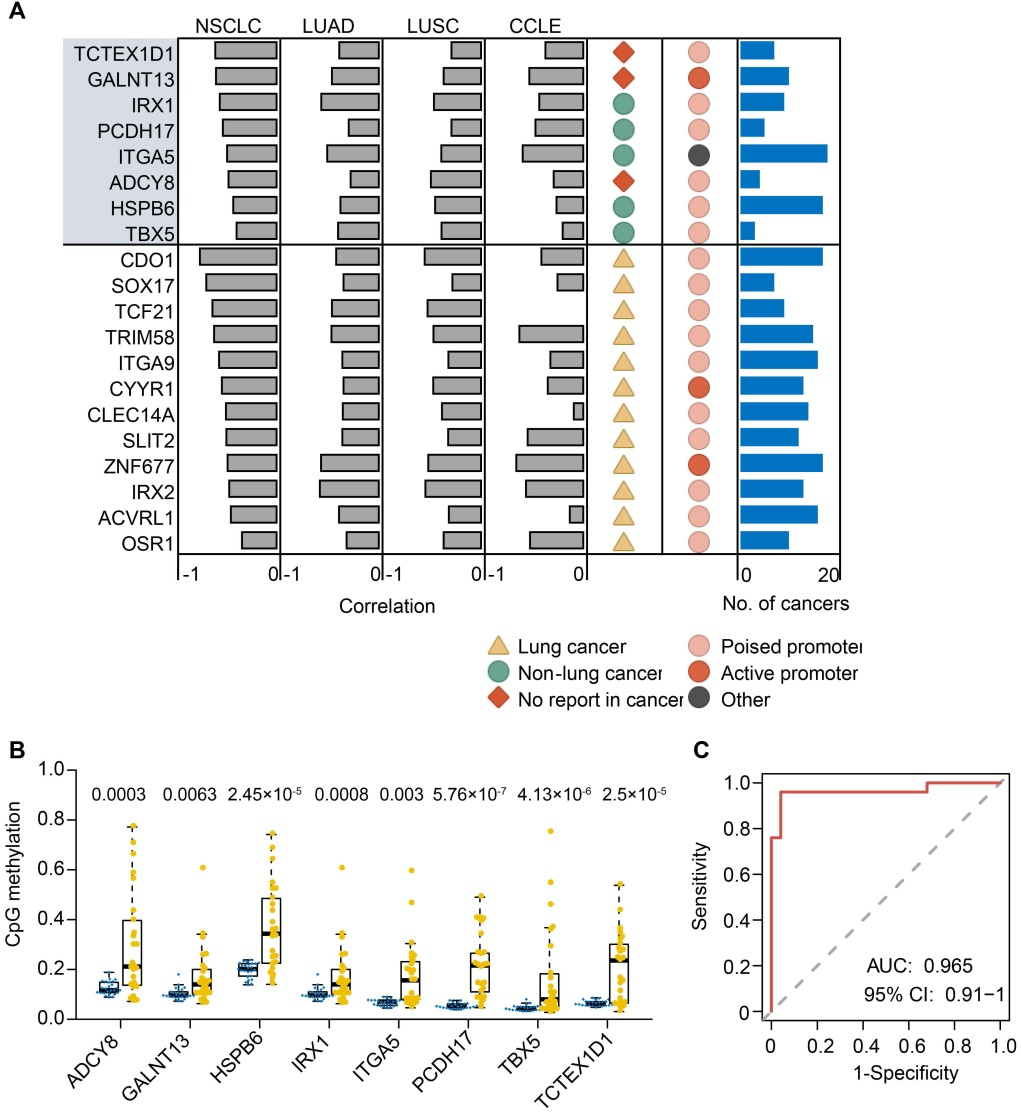
** Methylation-silenced driver genes in lung cancer.** (**A**) 20 methylation driver genes identified in lung cancer. Grey bars indicate the Spearman correlation calculated from our NSCLC data, TCGA LUAD and LUSC, and CCLE database. The dark yellow triangle indicates known methylation drivers in lung cancer, spring green circle indicates known methylation drivers in non-lung cancer, and the Indian red rhombus indicates first report in cancer. Light and dark red circles indicate genes pre-marked by poised and active promoter in ESCs, respectively. Number of cancer types from TCGA cohorts (blue bar) that detected the same methylation drivers is plotted in the right panel. (**B**) Validation of methylation changes of eight novel methylation driver genes in an independent cohort (23 paired tumor and normal tissues) by bisulfite pyrosequencing. The box indicates the median±1 quartile with each point representing one sample (yellow for tumors and blue for normal tissues). (**C**) ROC curve for random forest analysis using pyrosequencing value inputs of eight novel methylation biomarkers from an independent cohort. ROC analysis yielded an AUC of 0.965, showing high accuracy of distinguishing tumors from normal tissues.

The above data and previous reports suggested that the majority of methylated events were passenger methylation events which are probably a consequence of tumorigenesis 52, 53. Hence, we developed a heuristic strategy to pinpoint driver gene methylation events from passenger events in lung cancer. By integrative analysis of methylation levels of promoter DMRs (pDMRs; within the 2kb upstream and downstream of TSS and corresponding RNA-seq, this strategy identified putative methylation driver genes where not only both their transcript expression and methylation level were dysregulated in tumors, but also there were strong negative correlations between expression and methylation changes. As a result, we obtained a primary gene list in the discovery set, which consisted of 190 protein coding genes (FDR < 0.05, Spearman's rho < -0.3). To further reduce false positive discovery rates, we filtered the primary candidate gene list using the methylation and expression dataset of the lung cancer samples in TCGA cohorts including LUAD and LUSC. Finally, we obtained a high-confidence list with 31 unique genes including 20 hypermethylation drivers and 11 hypomethylation drivers that were recovered in both the LUAD and LUSC samples from TCGA cohorts, and a moderate-confidence list with 81 unique genes that were recovered in only one of the above cancer types (**Figure 5A** and**Table S8**). Here, we focused on the 20 hypermethylation driver genes in the high-confidence list for subsequent analysis.

Methylation status of the 20 hypermethylation driver genes showed a significant and highly negative correlation (FDR < 0.01, Spearman's rho < -0.3) with their expression (**Figure 5A**). We further analyzed the transcript expression and HM450 methylation data of 638 cancer cell lines from the CCLE database, and found that 15 of 20 hypermethylation driver genes exhibited a significant and highly negative correlation (FDR < 0.01, Spearman's rho < -0.3) between their expression and methylation in the promoter (**Figure 5A**), demonstrating reproducibility of the methylation driver list. We also examined such negative correlations for the 20 genes across 19 other cancer types in TCGA. We observed that 18 genes revealed a significant negative correlation (FDR < 0.01, Spearman's rho < -0.3) in at least five cancer types, except for ADCY8 and TBX5, which showed a cancer-type specific methylation driver. Moreover, the majority of methylation driver genes (16 of 20) belonged to ESC-poised marked genes (**Figure 5A**), further supporting our conclusion that preferential hypermethylation events in ESC-poised marked genes played a key role in tumorigenesis.

Twelve of 20 genes (60%) in the list were methylation driver genes described previously in lung cancer (**Figure 5A**), such as CDO1, SLIT2, SOX17 and TCF21. The remaining eight genes are newly identified methylation drivers in lung cancer, including 5 known cancer methylation driver genes in other cancer types (HSPB6, IRX1, ITGA5, PCDH17, TBX5) and 3 novel genes that had not been previously reported (ADCY8, GALNT13 and TCTEX1D1) (**Figure S7**). Furthermore, we verified the reproducibility of the methylation changes of eight novel methylation drivers in an independent set of 23 lung tumor and matched normal tissues using pyrosequencing (**Figure 5B**). All of them exhibited highly significant differences in methylation levels between tumor and normal tissues. Interestingly, the eight novel methylation drivers could distinguish lung tumors from normal samples with an area under the curve (AUC) of 0.965 (95%CI: 091-1) in an independent lung cancer cohort (**Figure 5C**), suggesting this eight-gene panel could be a potential diagnostic biomarker of lung cancer.

### Functional validation of novel methylation-silenced driver genes in lung cancer

**Figure 6:**
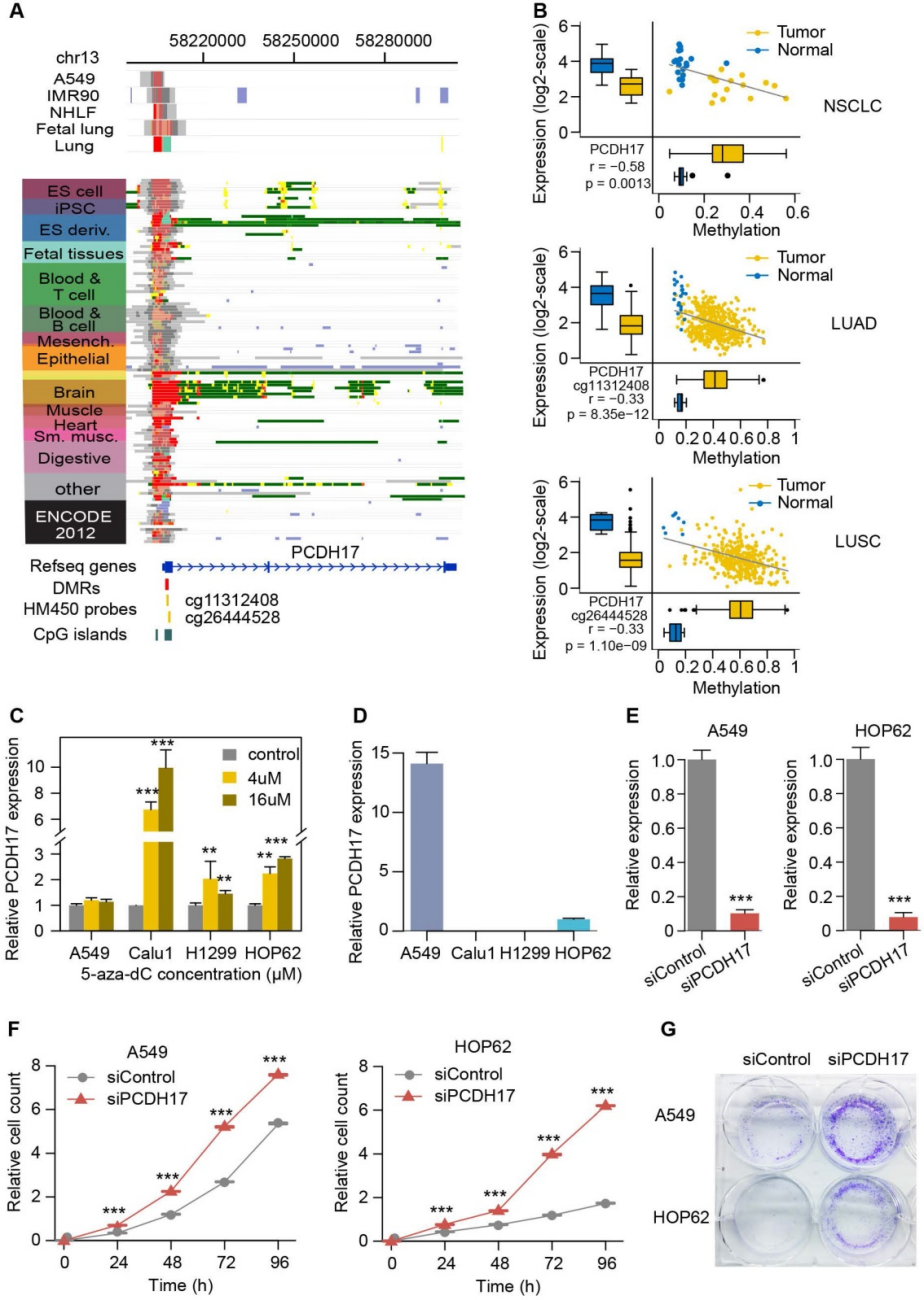
** Functional validation of PCDH17.** (**A**) The epigenomic chromatin profile of DMR in the PCDH17 promoter. Color-coded definitions of chromatin states are shown in Figure 2C. The position of DMR is highlighted by the red box. The CpG islands overlapped with the DMR are shown in a green box. The HM450 probes within the DMR are indicated by yellow lines. (**B**) Scatterplots and box plots of methylation of DMRs/CpGs within the PCDH17 promoter and expression of PCDH17 in RRBS NSCLC (top), TCGA LUAD (middle) and TCGA LUSC (bottom) cohorts. (**C**) Relative expression of PCDH17 after treatment with increasing concentration of 5-aza-dC in lung cancer cell lines A549, Calu1, H1299, and HOP62. (**D**) Relative expression of PCDH17 in lung cancer cell lines A549, Calu1, H1299, and HOP62. (**E**) Relative expression change of PCDH17 in A549 and HOP62 cells transfected with PCDH17 siRNA or control siRNA. (**F**) Growth curves of A549 and HOP62 cells transfected with PCDH17 siRNA or control siRNA. (**G**) Colony formation assays in A549 and HOP62 cells transfected with PCDH17 siRNA or control siRNA. The error bars indicate SD of three independent experiments. *P <0.05, **P <0.01, ***P <0.001 using a two-sided Student's t-test.

Considering that PCDH17 is a known tumor suppressor but not previously associated with lung carcinogenesis, we performed experimental investigation of its functional role in this tumor type. Its expression is higher in normal spleen, brain and lung, probably due to the active histone mark of their promoter in these tissues. However, for other tissue and cell types (e.g., ESCs and A549), their promoter regions are often targeted by a combination of active and repressive histone marks (poised chromatin state), resulting in lower expression in them (**Figure 6A** and **Figure S8**). The TSS of this gene is within CGI. Our analysis identified a 1,123 bp DMR (from 58,206,777 to 58,207,899 on chromosome 13) within this CGI, located at 124bp downstream of the gene's TSS. Two probes in HM450 from TCGA cohorts are also mapped to the DMR (**Figure 6A**). Increased methylation levels of both the probes and the DMR are significantly correlated with decreased gene expression in lung tumor compared to normal samples (**Figure 6B**). We further examined the consequence of demethylation on PCDH17 expression using the demethylating agent 5-aza-dC in four lung cancer cell lines (A549, Calu1, H1299, and HOP62). The expression of PCDH17 was found to be strongly upregulated in Calu1, H1299, and HOP62 cells (**Figure 6C**), suggesting that PCDH17 expression is regulated by promoter methylation. PCDH17 expression in A549 cells was the highest among the four cell lines, which probably explained why we did not observe significant expression change after 5-aza-dC treatment (**Figure 6D**). HOP62 also had the higher PCDH17 expression, thus the two cell lines were selected for inhibition of PCDH17 expression. The expression level of PCDH17 was substantially reduced (>85% and 90%, respectively) after small interfering RNA (siRNA) was transfected into cells (**Figure 6E**). Then clone formation assays and CCK-8 cell viability assays were performed to assess the effect of PCDH17 knockdown on A549 and HOP62 cell proliferation. The depletion of PCDH17 significantly promoted cell growth of A549 and HOP62 cells compared to cell lines with scrambled control (P < 0.001, **Figure 6F**). Downregulation of PCDH17 also strongly enhanced clone formation in both cell lines (**Figure 6G**). These findings indicate that PCDH17 suppresses lung cancer cell proliferation.

We also performed *in vitro* experiments to explore the functional role of other methylation drivers (IRX1, TBX5 and HSPB6) in lung cancer cell lines. We observed significant expression change of IRX1 and HSPB6 after 5-aza-dC treatment (**Figure S9A-B**). In addition, overexpression cell lines of IRX1, TBX5 and HSPB6 were successfully established (**Figure S9C-D**). Re-expression of IRX1 significantly inhibited lung cancer cells growth compared to control cells (**Figure S9E-F**). Cell migration was significantly reduced in lung cell lines with overexpression of IRX1, TBX5 and HSPB6 (**Figure S9G**). Overall, our results suggest that these newly identified methylation drivers play a tumor-suppressor role in lung tumor pathogenesis.

### Shared epigenetic control of ncRNAs and their host genes

**Figure 7:**
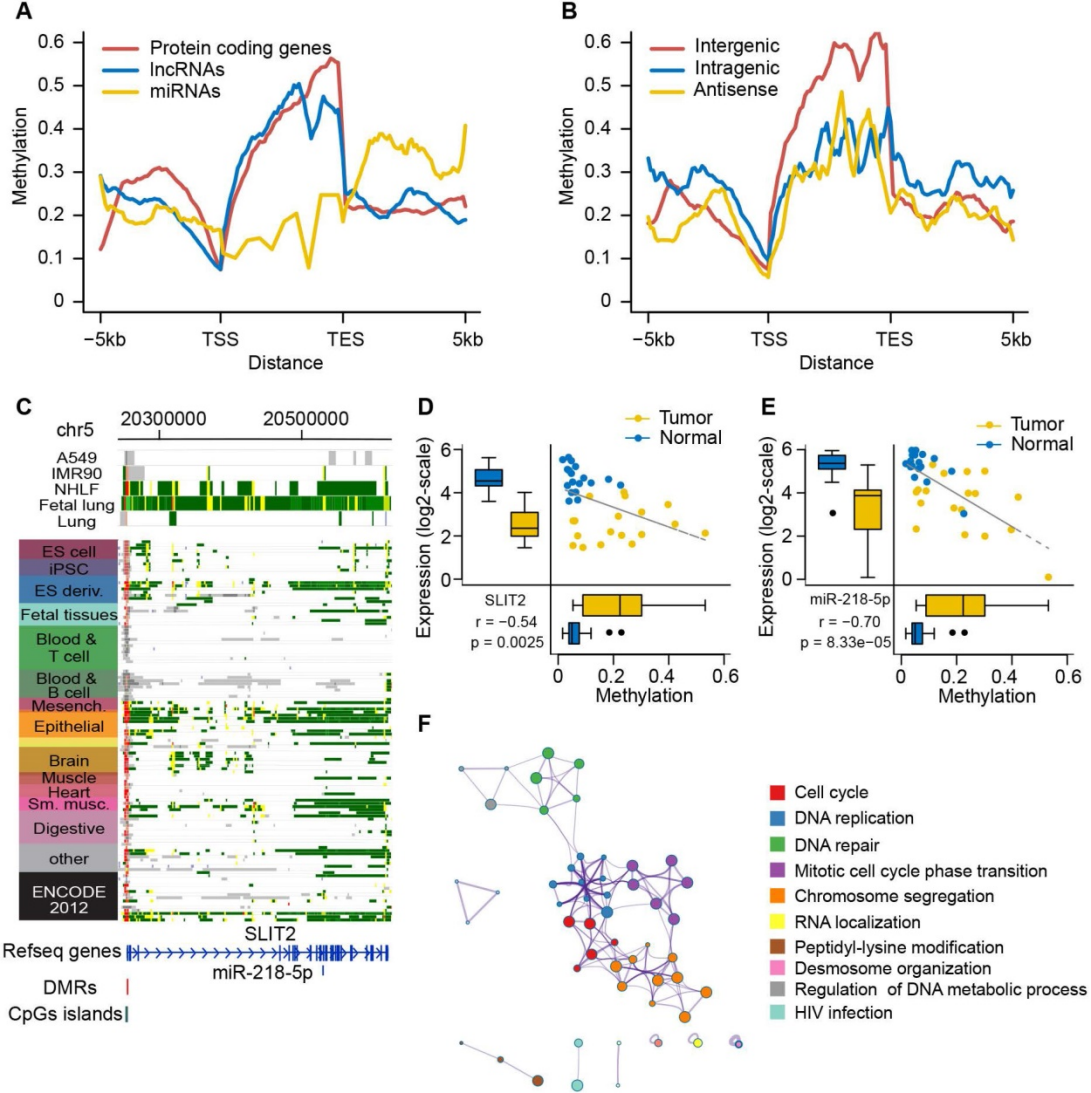
** DNA methylation alterations control expression of ncRNAs and their host genes. (A)** DNA methylation patterns of protein-coding genes and ncRNAs (lncRNAs and miRNAs) across the gene body and ±5kb flanking regions of the gene body**.** DNA methylation level is calculated according to 1kb windows with 100bp steps in lung normal samples.** (B)** DNA methylation patterns of three different subcategories of lncRNAs (intergenic, intragenic and antisense) across the gene body and ±5kb flanking the gene body.** (C)** The epigenomic chromatin profile of DMR in miR-218 and its host gene SLIT2 promoter. Color-coded definitions of chromatin states are shown in Figure 2C. The position of DMR is highlighted by the red box. The CpG islands overlapped with the DMR are shown in the green box.** (D)** Scatterplots and box plots of methylation of DMR within the SLIT2 promoter and its expression in the RRBS cohort.** (E)** Scatterplots and box plots of methylation of DMR within the promoter of SLIT2 and expression of miR-218 in the RRBS cohort.** (F)** The computational prediction, biological process that miR-218 is involved in.

Epigenetic alterations of ncRNAs have been reported to be involved in tumorigenesis in several cancer types 54, 55. Here, we focused on two common types of ncRNAs, lncRNAs and miRNAs. First, we evaluated the aberrant methylation pattern surrounding the TSSs and the transcript end sites (TESs) of protein coding genes, lncRNAs, and miRNAs in lung tumors and adjacent normal tissues. The depletion of DNA methylation in TSS and TES regions and the enrichment of DNA methylation in transcribed regions were observed in both protein coding genes and lncRNAs, but low levels of DNA methylation were distributed in miRNAs (**Figure 7A** and **Figure S10A**). We further investigated if three subtypes of lncRNAs (i.e., intergenic, intragenic and antisense), which were classified based on their relative position to nearby coding genes, had differential methylation profiles. More enrichment of DNA methylation toward the transcribed regions was exclusively observed in intergenic lncRNAs compared to the other types of lncRNAs (**Figure 7B** and **Figure S10B**). These results suggest that lncRNAs display similar methylation patterns with protein coding genes, but the three types of lncRNAs have distinct methylation profiles, which probably affect their regulatory role.

Moreover, we identified eight lncRNAs and five miRNAs showing significantly negative correlation between their methylation and expression (**Table S9**). Interestingly, three of them were co-regulated with their host genes by promoter hypermethylation, including miR-218 and its host gene SLIT2, miR-490 and its host gene CHRM2, and CDO1-LNC and its host gene CDO1 (**Figure 7C,** and **Figure S11A** and **12A**). miR-218 was recently reported to play a tumor suppressive role in lung cancer through regulation of the IL6/STAT3 pathway.

Additionally, based on the bioinformatics analysis of our corresponding miRNA-seq and RNA-seq data, miR-218 was downregulated in tumors and predicted to be involved in many important cancer-related biological processes, such as the cell cycle, DNA replication and DNA repair, further supporting the tumor suppressive role of miR-218 in lung cancer. However, how to regulate the expression of miR-218 remains elusive. Here, our findings suggest that promoter methylation is a probably selective mechanism of regulation of miR-218 and its host gene (**Figure 7D-F**). Epigenetic silencing of miR-490 and its host gene CHRM2 has been observed in gastric and colorectal cancer 56, 57. For the first time, we reported the potential epigenetic regulation of miR-490-3p and its host gene CHRM2 in NSCLC (**Figure S11B-D**).

CDO1-LNC, a lncRNA annotated in the GENCODE (v28), was also detected in our lung cancer data. It covered a stretch of 557bp within CDO1 (**Figure S12A**). In an effort to determine the potential functional role of CDO1-LNC, a Guilt-By-Association (GBA) analysis was performed, an analysis that has been widely used for studying lncRNAs 58. This analysis yielded a total of 1,975 protein coding genes that were significantly co-expressed with CDO1-LNC (FDR <0.01, Spearman's rho < -0.5 or >0.5). These genes were mainly enriched in the cell cycle, DNA replication and the development process, suggesting that it may be involved in tumorigenesis in NSCLC. In addition, hypermethylation of the same DMR is significantly correlated with expression of both CDO1-LNC and its host gene CDO1 (**Figure S12B-D**), indicating they are probably co-regulated by promoter methylation.

### Differential DNA methylation of transcription factor binding motifs in NSCLC

TF binding can be blocked by the methylation of TF binding sites in ESCs 59. Consequently, we sought to search for sets of TFs that modulate expression of our identified methylation driver genes in lung cancer. To ensure the power of identifying TFs, we focused on the primary gene list in the discovery set, which consisted of 190 methylation driver genes (**Table S8**). Then, 190 pDMRs in these genes were intersected with binding motifs of 84 TF groups obtained from the literature and de novo motif discovery in 427 human ChIP-seq datasets 60. Significant enrichment of motifs was observed in the hyperMe regions of lung tumor (46 in hyperMe regions *versus* 10 hypoMe regions; Fisher's test p value= 0.0002; **Table S10**). Finally, TFs that correspond to these enriched motifs were identified; the correlation between expression of the TFs and their targeted genes was calculated. As a result, 27 significantly correlated motifs in hyper-methylated and down-regulated genes, and eight in hypo-methylated and up-regulated genes were identified (FDR < 0.05; **Table S11**), including known methylation sensitive TFs such as SP1, YY1 and CTCF 61. For these TFs, the DNA methylation in their binding motifs regulated cancer-specific gene expression by affecting the binding ability of TFs in NSCLCs. The top ten highly significantly correlated motifs in hyper-methylated regions and the top five in hypo-methylated regions were selected for downstream analysis. At least one CpG site was observed in most of these selected motifs, further supporting that they were indeed involved in transcriptional regulation through CpG methylation states (**Figure S13**).

**Figure 8:**
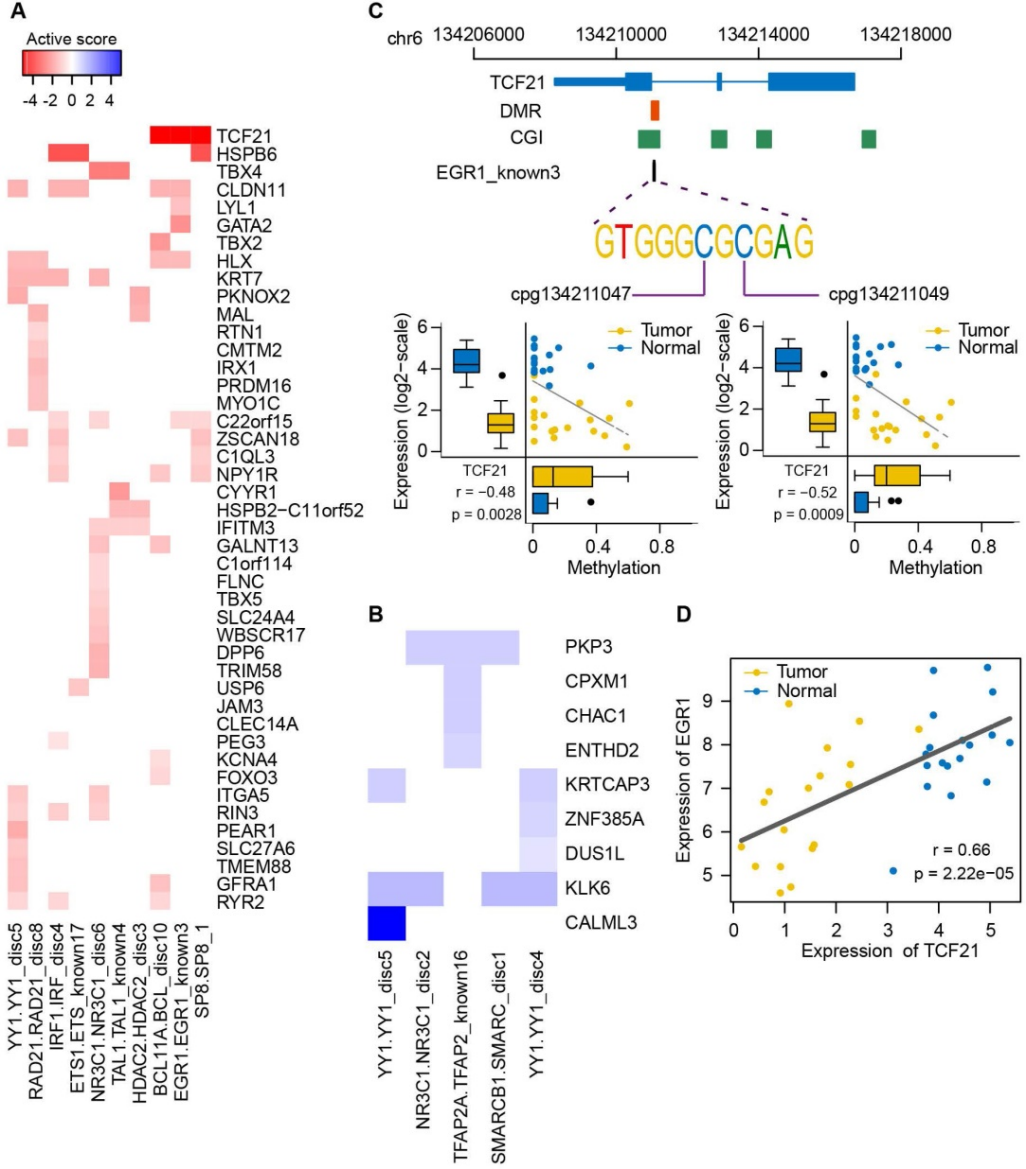
** Expression of epigenetically dysregulated genes is affected by methylation changes of transcription factor binding motifs in lung cancer. (A, B)** Activity plot integrating the correlation between methylation of transcription factor binding motifs and expressional fold changes of targeted methylation-driven genes. The strength of inactivation (red; A) and activation (blue; B) in tumors compared to normal samples is represented by color and intensity. **(C)** Correlation between methylation level of two CpG sites within the EGR1 binding motif of TCF21 and expression level of TCF21. Genomic coordinates of the EGR1 binding motif relative to TCF21 are shown in the top panel. **(D)** Correlation between EGR1 and TCF21 expression.

To determine which genes were strongly influenced by the binding vicinity of the corresponding TFs in NSCLC, we calculated activity scores by integrating the correlation of targeted gene expression with methylation level of pDMRs and expression level of TFs (**Figure 8A-B**). The strongly inactivated genes in lung tumors compared to normal samples, whose corresponding TF binding motifs were affected by DNA methylation, included well-known epigenetically dysregulated genes such as TCF21, CYYR1 and GATA2, as well as some novel epigenetically dysregulated genes such as HSPB6, ITGA5 and TBX5. The strongly activated genes in lung tumors were also found, including PKP3, a member of the armadillo protein family whose overexpression was a key feature in lung carcinogenesis 62. Of interest was the motif EGR1_known3 of EGR1, including two CpG sites (**Figure S13**). The genome-wide methylation level of this motif was lower than its neighbor regions, supporting that TF binding sites tend to lose methylation (**Figure S14**). The high variance of methylation in the motif and neighbor regions in lung tumors indicated that some CpG sites have been selectively methylated (**Figure S14**). EGR1, a zinc-finger tumor suppressor transcription factor, has been shown to regulate multiple tumor suppressors including TGFβ1, TP53 and PTEN 63. Here, we found the strongest inactivation of EGR1 binding motif within the TCF21, an epigenetically regulated tumor suppressor gene in lung cancer (**Figure 8A**). We found a significant association between the methylation level of the two CpG sites in the EGR1 binding motif and TCF21 expression (**Figure 8C**). The EGR1 expression was also highly correlated with expression level of TCF21 (**Figure 8D** and **Figure S15**). Moreover, it has been shown that TCF21 can be activated by WT1, recognizing and binding to EGR1-like motifs 64, 65. Taken together, EGR1 is potentially involved in transcriptional regulation of TCF21 and the methylation states of EGR1 binding motif may influence the ability of EGR1 or WT1 to bind to TCF21 in lung cancer.

## Discussion

Previous studies have explored the impact of DNA methylation events on lung tumor initiation and progression 19-22, 27-29, 66-68. However, those studies mainly investigated a single gene or a small number of genes and a NGS-based methylation investigation in lung cancer has not yet been reported. Here, we analyzed the genome-wide methylation profile in lung cancer using an enriched sequencing approach called RRBS. This NGS-based technique is greatly superior to traditional microarray-based methods with regard to its high CpG coverage, single-base resolution and low input requirement 30. In the present study, on average we could interrogate more than two million CpG sites in each sample for methylation analysis. Another major advantage of this method is the ability to search for DMRs spanning multiple consecutive CpG sites, which are robust and functionally relevant methylation events 47. Due to strong correlation among CpG sites within a genomic region of about 500bp, calling DMR could reduce the dimensionality of methylation data and thus increase the power and robustness of identifying differential methylation events, especially for low-coverage regions. From our RRBS data, we uncovered 4,410 hypermethylated and 4,824 hypomethylated DMRs using the circular binary segmentation algorithm, which could help us better dissect the casual role of DNA methylation in lung cancer.

As expected, we observed widespread aberrations of DNA methylation in our lung cancer data including global DNA hypomethylation and CGI-specific hypermethylation, which is largely in line with previous reports in several malignancies 44, 69, suggesting their important roles in tumorigenesis. Based on the top two principal components of the PCA results, we found that methylation profiles in tumor specimens were largely different from each other, which could partially explain intra-tumor heterogeneity, whereas their corresponding normal counterparts presented similar methylation landscapes. Interestingly, the methylation level of our identified hyperMe-DMRs remained significantly higher in tumor tissues from 21 cancer types of TCGA and the 1,028 CCLE cell lines compared to that in corresponding normal tissues and cells, indicating that hypermethylation of these regions is cancer specific. Additionally, 46.1% of the identified hyperMe-DMRs in lung cancer were overlapped with poised promoters in ESCs. This further confirmed that the frequent methylation of polycomb targets is a hallmark of lung cancer and many other human cancer types 48, 49. However, our observations also suggest that methylation silencing of these genes is a highly selective pressure during tumor formation and potentially affects tumor pathogenesis, rather than a residual stem-cell memory. Strikingly, 80% of our identified hypermethylation driver genes were pre-marked with a poised promoter in ESCs.

It is challenging to pinpoint cancer driver events from a large number of DNA methylation changes in genome-wide methylation studies. In the present study, we integrated DNA methylation with matched gene expression from our own lung cancer data and data provided by TCGA cohorts. Using this strategy, we identified 20 hypermethylation driver genes, of which 12 were recovered from previous studies and known epigenetic-regulated genes (e.g., SLIT2, CDO1 and TCF21), suggesting that the integration of DNA methylation with other types of omics data, such as RNA-seq, is an effective method to study methylation regulation of genes. Moreover, all of the eight novel hypermethylation driver genes (PCDH17, IRX1, ITGA5, HSPB6, TBX5, ADCY8, GALNT13 and TCTEX1D1) were successfully validated in an independent cohort via a pyrosequencing approach, with an AUC of 0.965 for prediction of lung cancer patients. This indicates that these novel drivers could be used as promising diagnostic biomarkers in the clinic. Notably, they have not been included in the 14 currently commercially used DNA methylation-based biomarkers 70.

Considering the recapitulation of previously published cancer driver genes, we experimentally tested the tumor-suppressive role of four new methylation driver genes in lung cancer cells, including PCDH17, IRX1, HSPB6 and TBX5, which are known driver genes but not linked with lung tumorigenesis 71-78. Expression of these genes was upregulated upon treatment with 5-Aza-dC demethylation, suggesting that their expression is regulated by DNA methylation. Notably, the putative methylation driver PCDH17 was previously described as a tumor suppressor that induces tumor cell apoptosis and autophagy, and was functionally hypermethylated in gastric, colorectal, urological and esophageal cancers 76-78. We also found that inhibition of PCDH17 promotes cell proliferation, suggesting its tumor-suppressive role in lung cancer. In addition, we validated the tumor-suppressive role of IRX1, TBX5 and HSPB6 in lung cancer. Of note, our identified methylation drivers are not within the gene list of 299 cancer driver genes that were identified by PanSoftware using somatic mutations and insertions/deletions in pan-cancer data and represented the largest discovery of cancer genes thus far 79. This suggests that analysis of methylation events could help to identify additional drivers and provide important research candidates.

Epigenetic regulation of ncRNAs in cancer have been recently characterized 80. Using the same data mining strategy described above, we also discovered 13 methylation driver ncRNAs (i.e., eight lncRNAs and five miRNAs) in lung cancer. This suggests that epigenetic alteration is a selective mechanism for regulation of ncRNA expression in cancer. Strikingly, our observations showed that several ncRNAs and their host genes were co-regulated by the same DMRs within the promoter, such as miR-218 and its host gene SLIT2, miR-490 and its host gene CHRM2, and CDO1-LNC and its host gene CDO1. This offers the possibility of developing more effective epigenetic therapeutic drugs to target the common DMRs that impact ncRNAs and the host genes, both of which play a critical role in tumor initiation and progression.

It has been demonstrated that the binding affinity of some TFs to DNA motifs is affected by CpG methylation status *in vivo* 81. A recent study suggested that loss of NRF1 binding was caused by local hypermethylation 82. Based on this regulatory paradigm, we correlated DNA methylation and expression level of targets with expression level of enriched TFs to identify cancer-specific TFs in lung cancer at a large scale. Using this method, we discovered several TFs and their target genes. Notably, we uncovered a potential regulatory relationship between EGR1 and TCF21. The strong correlation between methylation level of two CpG sites within EGR1 binding motif and TCF21 expression suggests that EGR1 transcriptionally regulates TCF21 expression through an epigenetic mechanism. Our observations also showed that the underlying connection between TFs and DNA methylation may be complex. For example, several DNA binding motifs of TFs did not span CpG sites, such as NR3C1_disc6, NR3C1_disc2 and SMARC_disc1 (**Figure S13**). However, the methylation level of neighborhood regions flanking these DNA motifs showed a strong correlation with expression of these TFs as well as target genes. Similar findings were reported in previous studies in which the binding ability of OCT4 was sharply reduced providing that DNA methylation occurred within 100 bp on each side of the targeted motif of OCT4, but no CpG sites were located within its recognized motifs 83, 84. Therefore, the neighborhood regions of TF binding sites may also play a role in gene regulation and should not be overlooked in future studies. Further characterization and experiment-based validation of the identified regulators may lead to the discovery of novel epigenetically dysregulated pathways in lung cancer and give new insights into the function of DNA methylation alterations in cancer.

## Conclusions

Our findings demonstrated that RRBS is an effective and robust approach to explore the cancer methylome. In our study, we discovered a large number of hypermethylation events pre-marked by poised promoter in ESCs, being a hallmark of lung cancer. These hypermethylation events showed a high conservation across cancer types and cancer cell lines. Among them, we identified and experimentally confirmed a group of methylation driver genes (e.g., PCDH17, IRX1, TBX5 and HSPB6) that play a critical role in neoplasia initiation, promotion, and progression. We also revealed shared epigenetic control of ncRNAs and their host genes that are controlled by the same promoter hypermethylation. Furthermore, we detected sets of TF regulators driving the expression of epigenetically dysregulated genes, guiding us to investigate the potential novel pathways and mechanisms by which DNA methylation regulates gene expression in lung cancer. Our study also provides a valuable resource for future efforts to identify DNA methylation-based diagnostic biomarkers, develop cancer epigenetic therapy and study cancer pathogenesis.

## Supplementary Material

Supplementary figures and tables.

## Abbreviations

AUC: an area under the curve
CCLE: Cancer Cell Line Encyclopedia
CGI: CpG island
DMR: differentially methylated regions
ENCODE: Encyclopedia of DNA Elements
ESCs: embryonic stem cells
FDR: false discovery rate
GO: gene ontology
hyperMe-DMRs: hypermethylated DMRs
hypoMe-DMRs: hypomethylated DMRs
lncRNAs: long noncoding RNAs
LUAD: lung adenocarcinoma
LUSC: lung squamous cell carcinomas
ncRNAs: noncoding RNAs
NGS: next-generation sequencing
NSCLC: non-small cell lung cancer
PCA: principal component analysis
pDMRs: promoter DMRs
ROC: receiver operating characteristic
RPKM: reads per kilobase of exon per million fragments mapped
RPM: Reads Per Million
RRBS: reduced representation bisulfite sequencing
siRNA: small interfering RNA
TCGA: The Cancer Genome Atlas
TES: transcript end sites
TF: Transcription factor
TSS: transcript start site
