# Supplementary material for: An integrated epigenomic-transcriptomic landscape of lung cancer reveals novel methylation driver genes of diagnostic and therapeutic relevance
Source: Theranostics. 2021 Mar 11;11(11):5346–64. doi: 10.7150/thno.58385 (PMC8039961; doi:10.7150/thno.58385)
Supplement: Supplementary file 1 — Supplementary figures and tables. [file thnov11p5346s1.zip › RRBS-Suppl-Methods-and-Figure-S1-S15-R1.pdf]

An integrated epigenomic–transcriptomic landscape of lung cancer reveals novel methylation driver genes of diagnostic and therapeutic relevance

Sun *et al.*

Supplementary Materials

Supplementary Methods

Supplementary Tables (Tables S1 to S11 in the format of Excel files can be downloaded separately).

Table S1. Characteristics of patient samples used in the study.

Table S2. Sequencing quality of RRBS (A), RNA-seq (B) and miRNA-seq (C).

Table S3. Summary of TCGA samples used in this study.

Table S4. A list of primers used for pyrosequencing validation of eight novel methylation driver genes in an independent samples.

Table S5. siRNA and qPCR primers used in the vitro assays.

Table S6. Summary of 4,810 hyperMe-DMRs (A) and 4,824 hypoMe-DMRs (B).

Table S7. Over-represented categories of molecular signature.

Table S8. Methylation driver genes identified in the study. (A) Confirmed in both TCGA LUAD and TCGA LUSC cohorts, (B) Confirmed only in TCGA LUAD cohort, (C) Confirmed only in TCGA LUSC cohort, (D) Not confirmed in TCGA LUAD cohort or TCGA LUSC cohort.

Table S9. Methylation driver ncRNAs identified in the study

Table S10. Enrichment of transcription factor binding motifs in hypermethylated and down-regulated methylation-driven genes (A) and in hypomethylated and up-regulated methylation-driven genes (B).

Table S11. Transcription factors significantly correlated with hypermethylated and down-regulated methylation-driven genes (A) and hypomethylated and up-regulated methylation-driven genes (B).

Supplementary Figures (Figures S1 to S15 are presented below)

Supplementary Methods

Library preparation for RNA-seq

Transcriptome analysis of these lung tumor and adjacent normal tissues was done using RNA-seq as we described previously [1]. Briefly, total RNA was isolated using TRIzol according to the manufacturer's instructions (Invitrogen). cDNA libraries were prepared using the TruSeq RNA Sample Preparation Kit (Illumina). Libraries were quantified by quantitative (q)PCR according to the Illumina's qPCR quantification guide to ensure uniform cluster density. Samples were multiplexed six per lane on a 300 Gb flow cell and paired-end sequenced with an Illumina HiSeq 2000.

Library preparation for small RNA sequencing

Small RNA libraries were prepared with the TruSeq Small RNA Sample Preparation Kit from Illumina with modifications [2]. In brief, 100 ng of total RNA were ligated with 3' and 5' adapters and reverse-transcribed to cDNA by SuperScript II reverse transcriptase. The cDNA was amplified with 15 cycles of PCR during which index sequences were incorporated. Libraries were validated with a DNA 1000 chip in an Agilent 2000 bioanalyzer. Twelve indexed libraries per lane were sequenced in an Illumina HiSeq 2000 sequencer.

siRNA transfection and cell proliferation assay

A549 and HOP62 cells were transfected with synthesized siPCDH17 (**Table S5**) or negative siRNA using 6-well plates with GeneMute^{TM} reagent for 24 h. Cell proliferation was determined using the cell counting kit-8 (Dojindo Laboratories, Kumamoto, Japan). In brief, cells were transfected with siRNAs using GeneMute^{TM} reagent (SignaGen^{TM} Laboratories); 24h after transfection, approximately 2,000 cells were placed into each well of 96-well plates. CCK-8 solution was added at 0 h, 24 h, 48 h, 72 h and 96 h after cell placement. After adding 10ul CCK-8 solution, cells were incubated for 1.5 h at 37 °C. The absorbance was measured at 450nm.

Overexpressed vector construction and transfection

TBX5, IRX1 and HSPB6 were amplified by polymerase chain reaction (PCR) from Hela cell cDNA and subcloned into the BamHI/XhoI site of the pENTER expression vector. Then constructed vectors were transfected into lung cancer cells. In brief, A549 and H1299 cells were maintained in RPMI Medium 1640 basic (1X) containing 10% FBS. A549 and H1299 cells were plated at a density of 2.0×10^4 cells per well in a 6-well plate for 24 h. Cells were then transfected with 1.5μg of total DNA together with 3.75ul of Lipofectamine^{TM} 3000 reagent (Life) per well. Cells were subjected to in vitro studies 24 hours after transfection. qPCR primers used in the study can be found in **Table S5**.

Soft agar clone formation assay

Lung cancer cells were transfected with siRNA or overexpressing vector or negative controls for 24 h. Then, 1,500 cells were plated in 6-well culture plates and cultured in RPMI 1640 medium supplemented with 10% FBS for one week. These cells were then fixed with methanol and stained with crystal violet solution.

Migration assay

In vitro migration and invasion assays were performed using transwell chambers. Lung cancer cells were transfected with siRNA or overexpressing vector or negative controls for 24 h. Then these cells were cultured with serum-free RPMI 1640 for 24 h. These cells were then detached and resuspended in serum-free RPMI 1640 medium. Next, 3×10^4 cells in 300ul cell suspensions were added in the upper transwell chambers for migration assay or the upper transwell chambers coated with Matrigel for invasion assay, and the RPMI 1640 with 10% FBS was added to the bottom chamber. Migrated and invasive cells were stained with 0.1% crystal violet. Images were captured from each membrane and the number of migrations was counted under a microscope.

References:

1. Liu Y, Liu P, Yang C, Cowley AW, Jr., Liang M: **Base-resolution maps of 5-methylcytosine and 5-hydroxymethylcytosine in Dahl S rats: effect of salt and genomic sequence.** *Hypertension* 2014, **63:**827-838.

2. Kriegel AJ, Liu Y, Liu P, Baker MA, Hodges MR, Hua X, Liang M: **Characteristics of microRNAs enriched in specific cell types and primary tissue types in solid organs.** *Physiol Genomics* 2013, **45:**1144-1156.

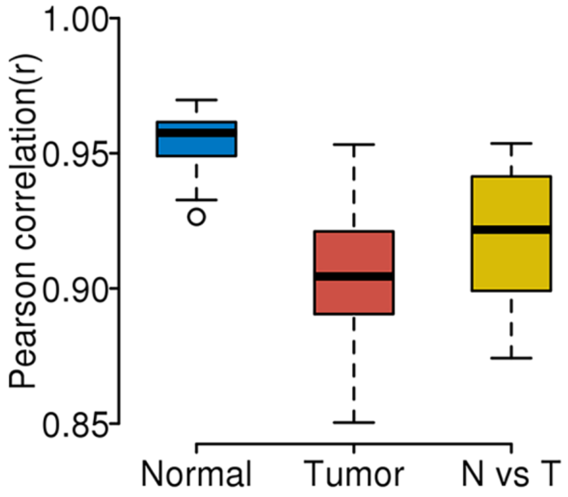

Figure S1. Correlations between RRBS samples. Box plots of Pearson correlation coefficients calculated among 18 normal samples, 18 tumor samples, and 18 normal/tumor pairs, respectively. N vs. T indicates normal/tumor pairs.

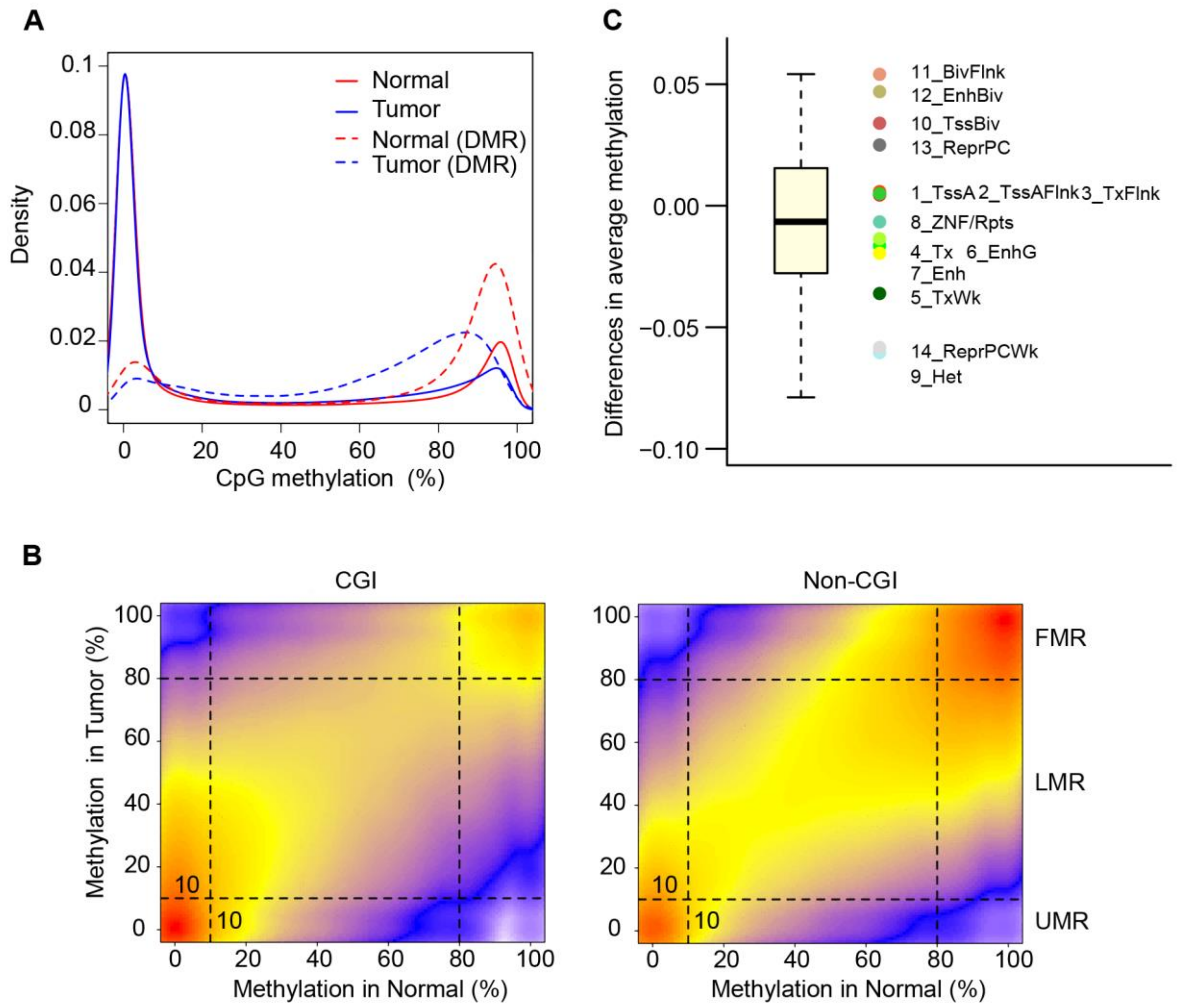

Figure S2. Characteristics of the methylation level of CpG sites across lung tumors and adjacent normal tissues. (**A**) Density plots of average methylation level of CpGs from the background set (n = 2,166,853; solid lines) and DMRs (n = 162,603; dashed lines) across normal or tumor samples, respectively. (**B**) Correlation between average methylation level of tumor and normal samples in CpG islands (CGI; left) and outside CpG islands (non-CGI; right). Orange indicates high density of CpG sites and purple indicates low density. UMR, 0–10%; LMR, 10%–80%; FMR, 80%–100%. (**C**) Average methylation differences (average for tumor samples vs. normal samples) across chromatin segments annotated in ESCs.

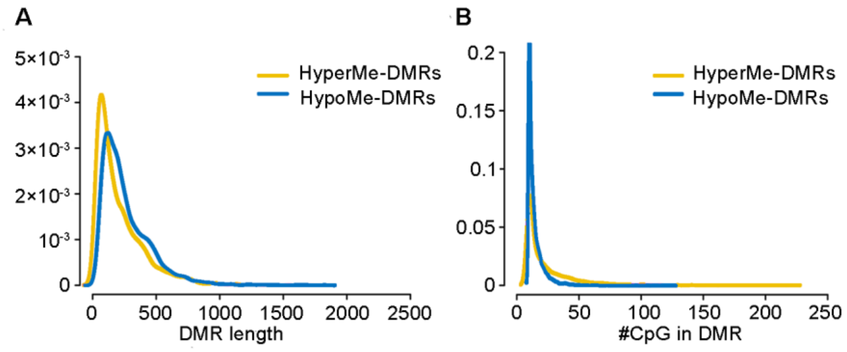

Figure S3. Length of DMRs and number of CpG sites in DMRs. (**A**) Distribution of the length of hypermethylated and hypomethylated DMRs. (**B**) Distribution of the number of CpG sites in hypermethylated and hypomethylated DMRs. HyperMe-DMRs, hypermethylated DMRs; HypoMe-DMRs, hypomethylated DMRs.

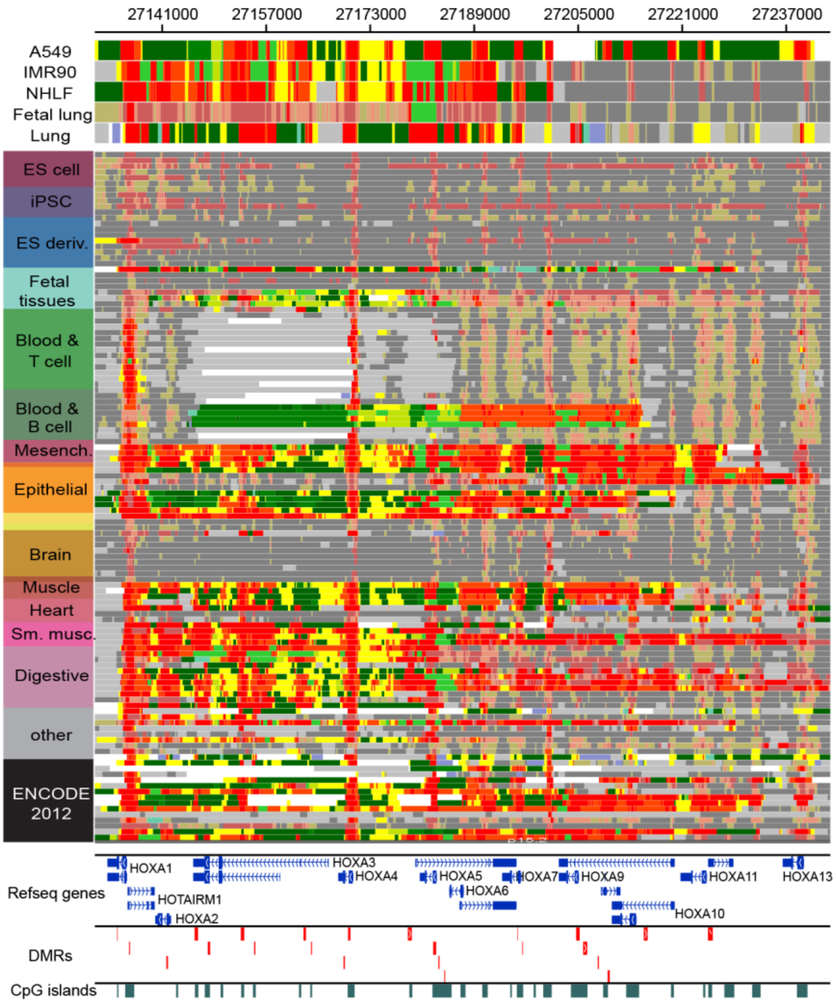

Figure S4. The epigenomic chromatin profile of DMR in HOXA cluster genes. Color-coded definitions of chromatin states are shown in Figure 2C. The position of the DMR is highlighted by the red box. The CpG islands overlapped with the DMR are shown in the green box.

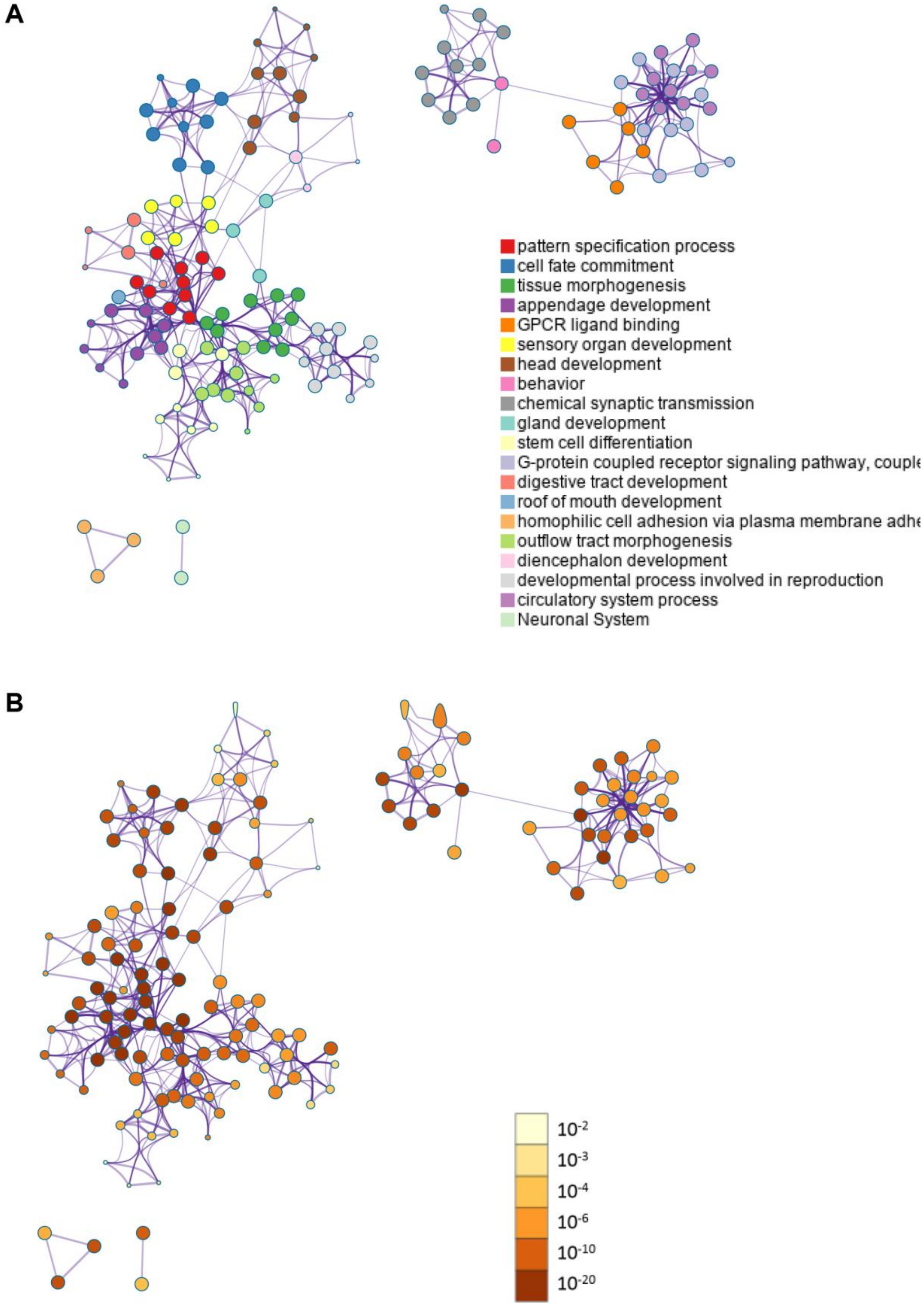

Figure S5. Gene ontology analysis of genes with HyperMe-DMRs in their promoters. (**A**) Enriched gene ontology (GO) term. (**B**) Statistical significance of corresponding GO term.

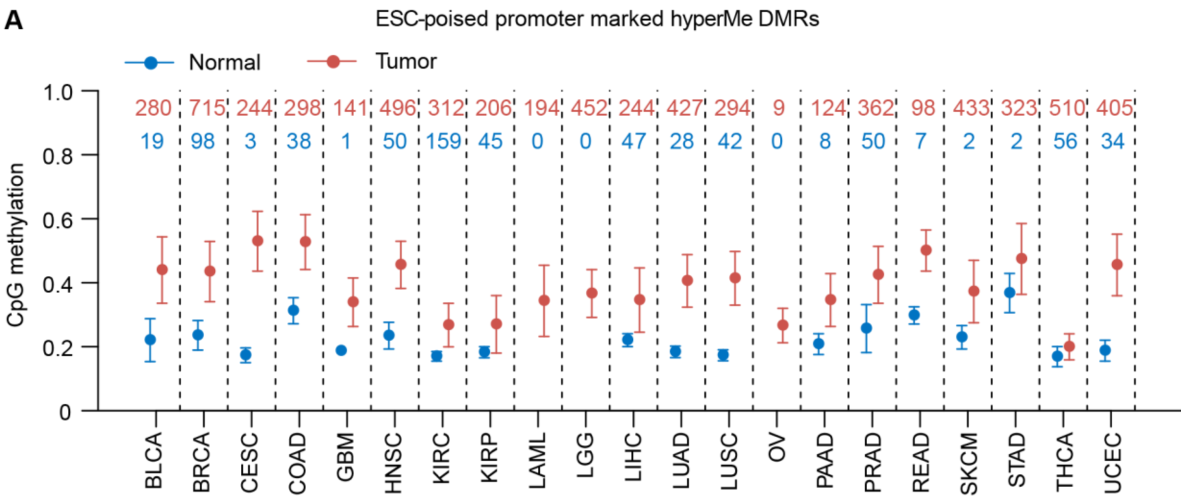

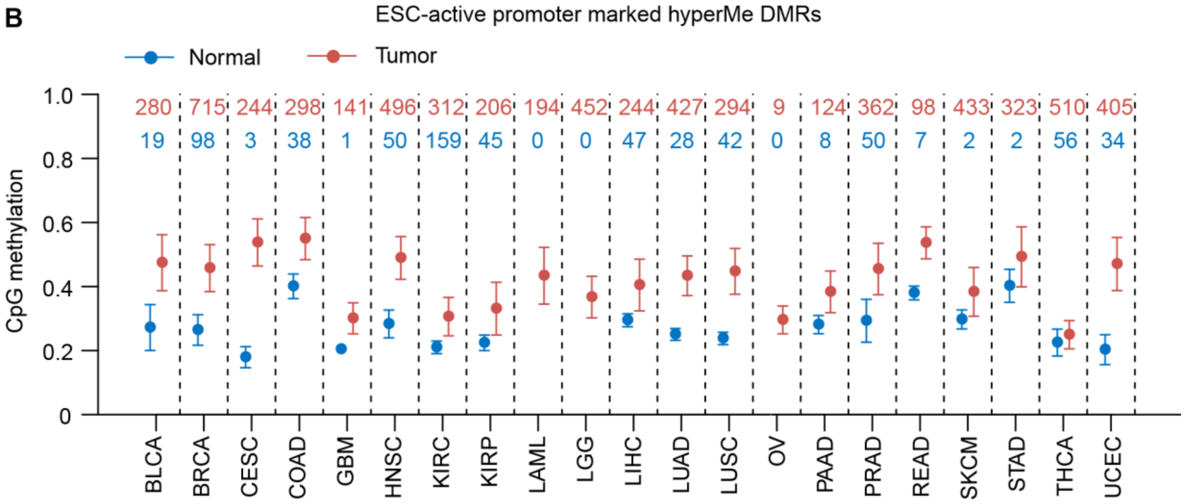

Figure S6. Methylation levels of hyperMe-DMRs pre-marked by poised promoter (A) and active promoter (B) in ESCs between tumor and normal samples across 21 cancer types. Data are shown as mean ± SD. The top digits signified number of tumor (red) and normal (green) samples analyzed. SD, standard deviation.

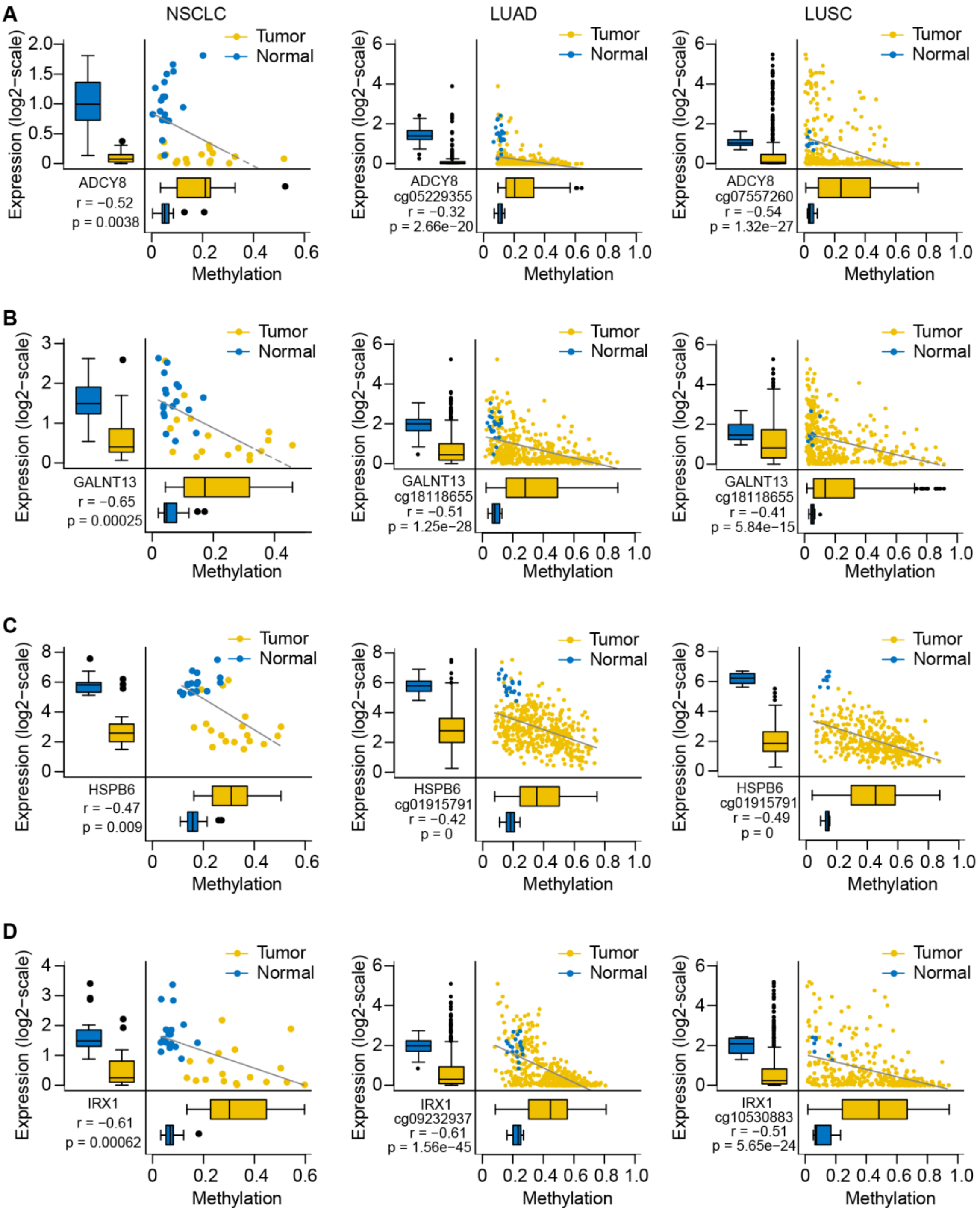
A
NSCLC
LUAD
LUSC
Tumor
Normal
Expression (log2−scale)
Methylation
ADCY8
r = −0.52
p = 0.0038
ADCY8
cg05229355
r = −0.32
p = 2.66e−20
ADCY8
cg07557260
r = −0.54
p = 1.32e−27
B
GALNT13
r = −0.65
p = 0.00025
GALNT13
cg18118655
r = −0.51
p = 1.25e−28
GALNT13
cg18118655
r = −0.41
p = 5.84e−15
C
HSPB6
r = −0.47
p = 0.009
HSPB6
cg01915791
r = −0.42
p = 0
HSPB6
cg01915791
r = −0.49
p = 0
D
IRX1
r = −0.61
p = 0.00062
IRX1
cg09232937
r = −0.61
p = 1.56e−45
IRX1
cg10530883
r = −0.51
p = 5.65e−24

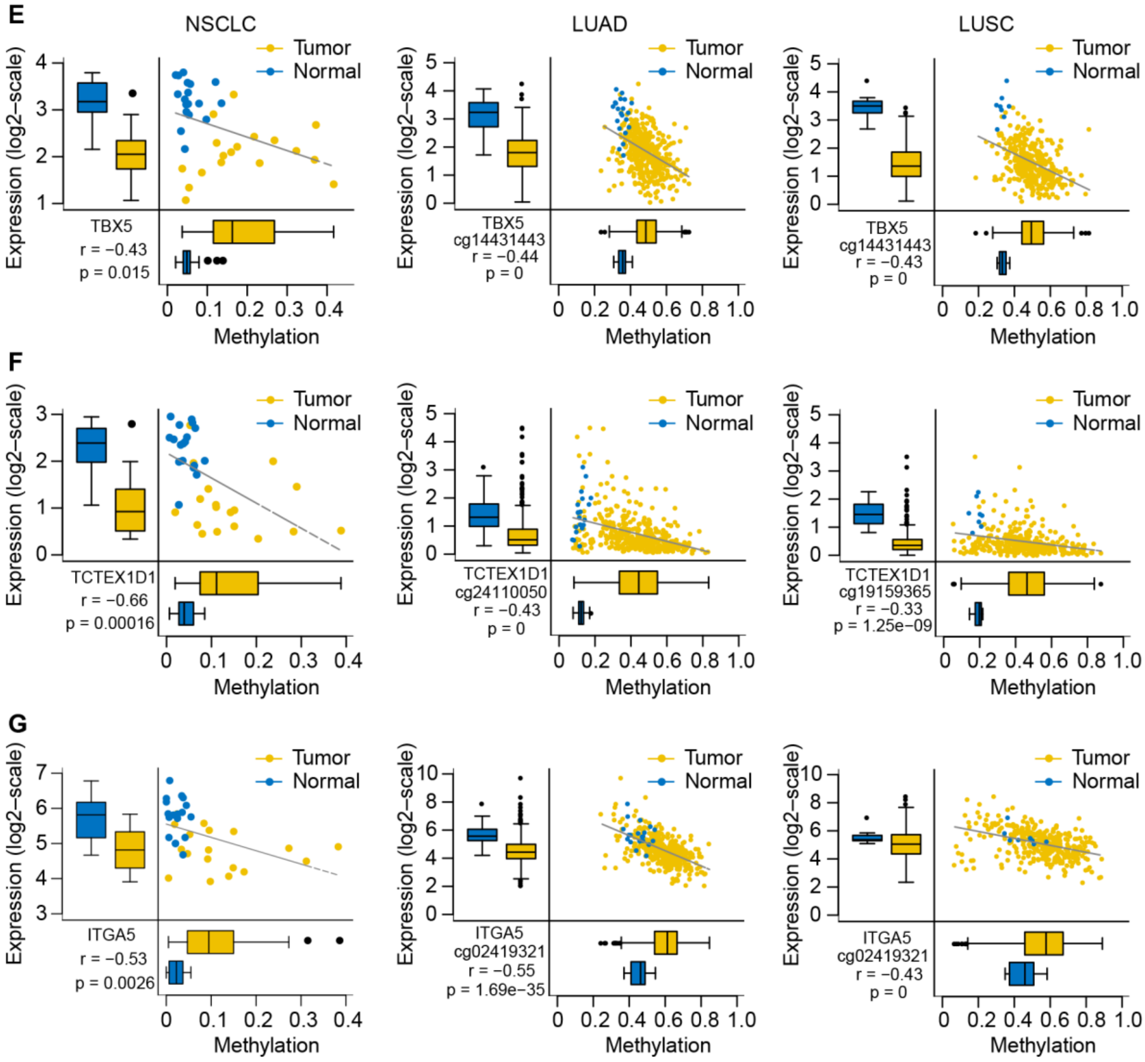

Figure S7. Scatterplots and box plots expression of novel methylation driver genes and methylation of DMR/CpGs within their promoters in RRBS NSCLC, TCGA LUAD and TCGA LUSC cohorts. (**A**) ADCY8, (**B**) GALNT13, (**C**) HSPB6, (**D**) IRX1, (**E**) TBX5, (**F**) TCTEX1D1, (**G**) ITGA5.

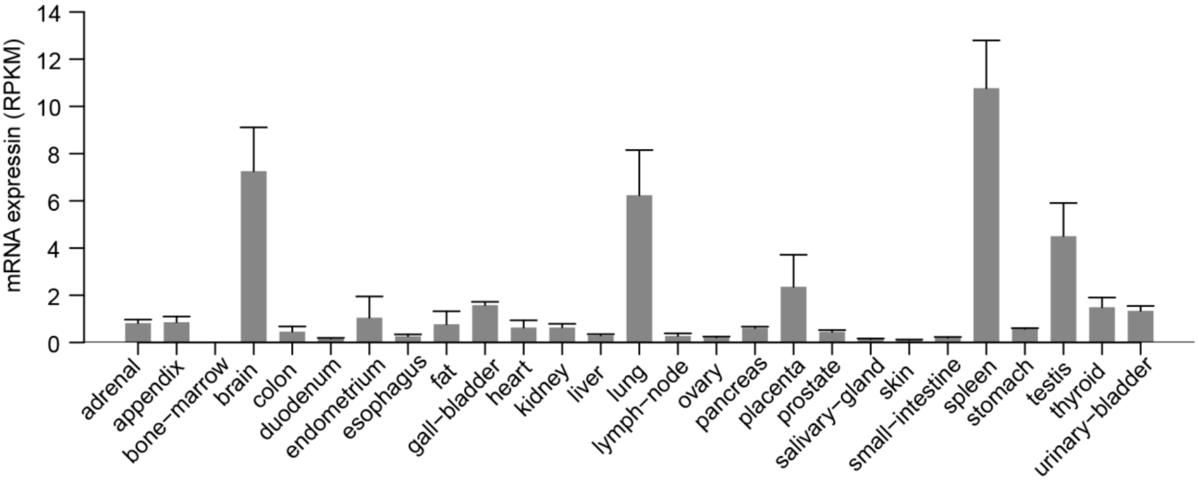

Figure S8. mRNA expression of PCDH17 across tissue samples from 95 human individuals representing 27 different tissues. Data were downloaded from the Publication: PMID 24309898.

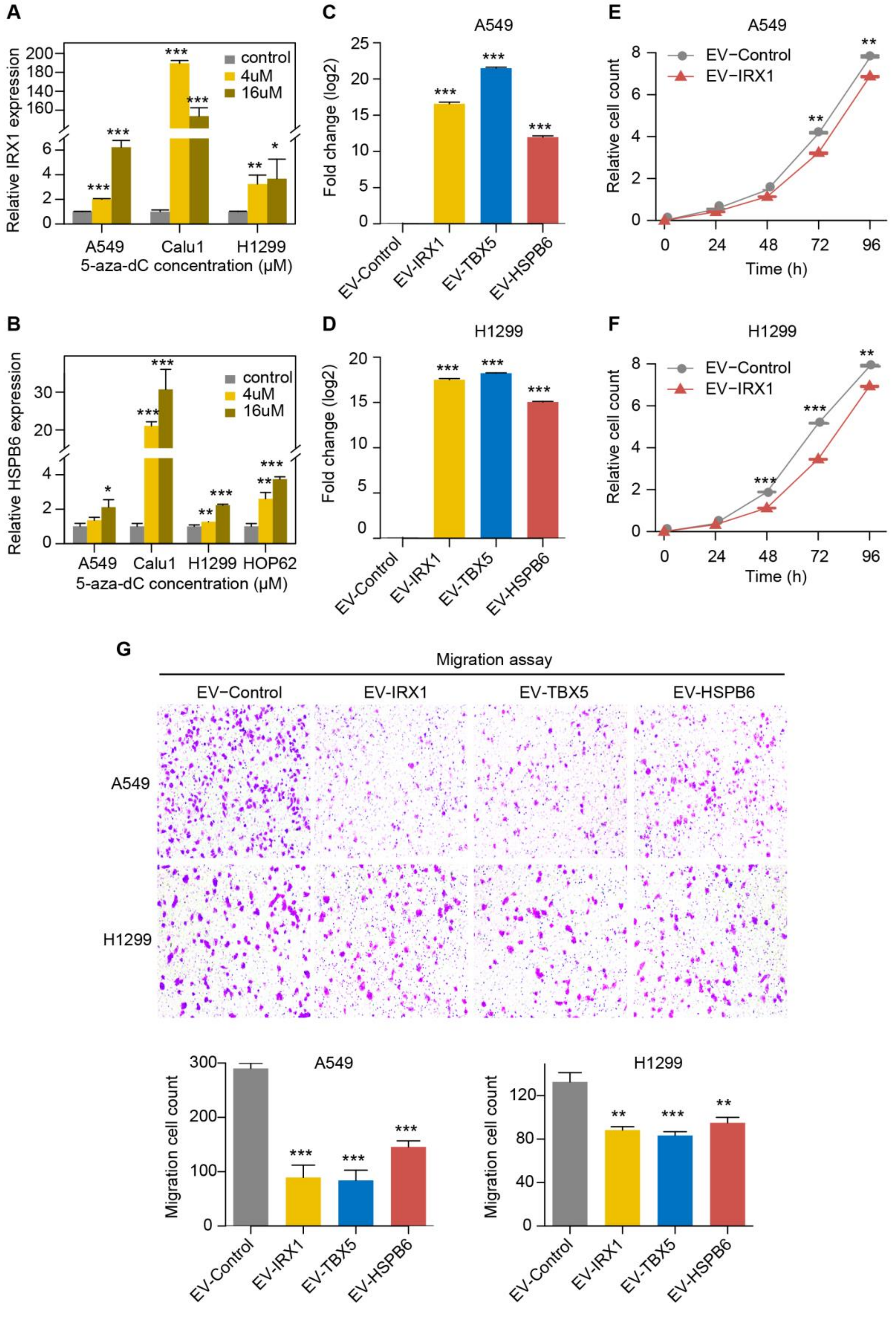

Figure S9. Functional validation of IRX1, TBX5, and HSPB6. (**A**) Relative expression of IRX1 after treatment with increasing concentration of 5-aza-dC in lung cancer cell lines

A549, Calu1, and H1299. (**B**) Relative expression of HSPB6 after treatment with increasing concentration of 5-aza-dC in lung cancer cell lines A549, Calu1, H1299 and HOP62. (**C-D**) Relative expression change of IRX1, TBX5 and HSPB6 in A549 (**C**) and H1299 (**D**) cells transfected with empty or IRX1, TBX5 and HSPB6 overexpression vectors. (**E-F**) Growth curves of A549 (**E**) and H1299 (**F**) cells transfected with empty or IRX1 overexpression vectors. (**G**) Migration assay following overexpression of IRX1, TBX5, and HSPB6 in A549 and H1299 cells. The error bars indicate SD of three independent experiments. *P <0.05, **P <0.01, ***P <0.001 using a two-sided Student's t-test.

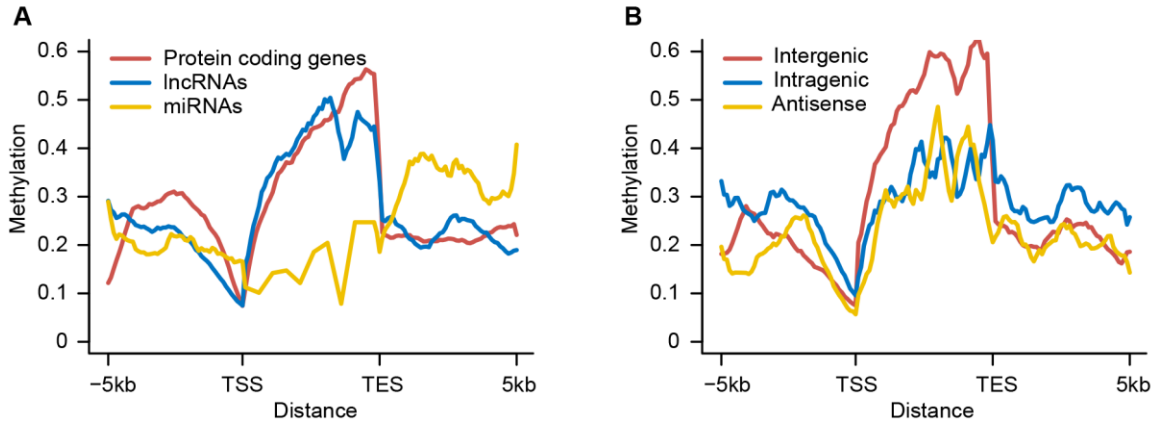

Figure S10. **DNA methylation patterns across the gene body and ±5kb up- or down-stream of gene body in lung tumors.** (**A**) DNA methylation patterns of protein-coding genes and ncRNAs (lncRNAs and miRNAs). (**B**) DNA methylation patterns of three different subcategories of lncRNAs (intergenic, intragenic and antisense). DNA methylation level is calculated according to 1kb windows with 100bp steps in lung tumor samples.

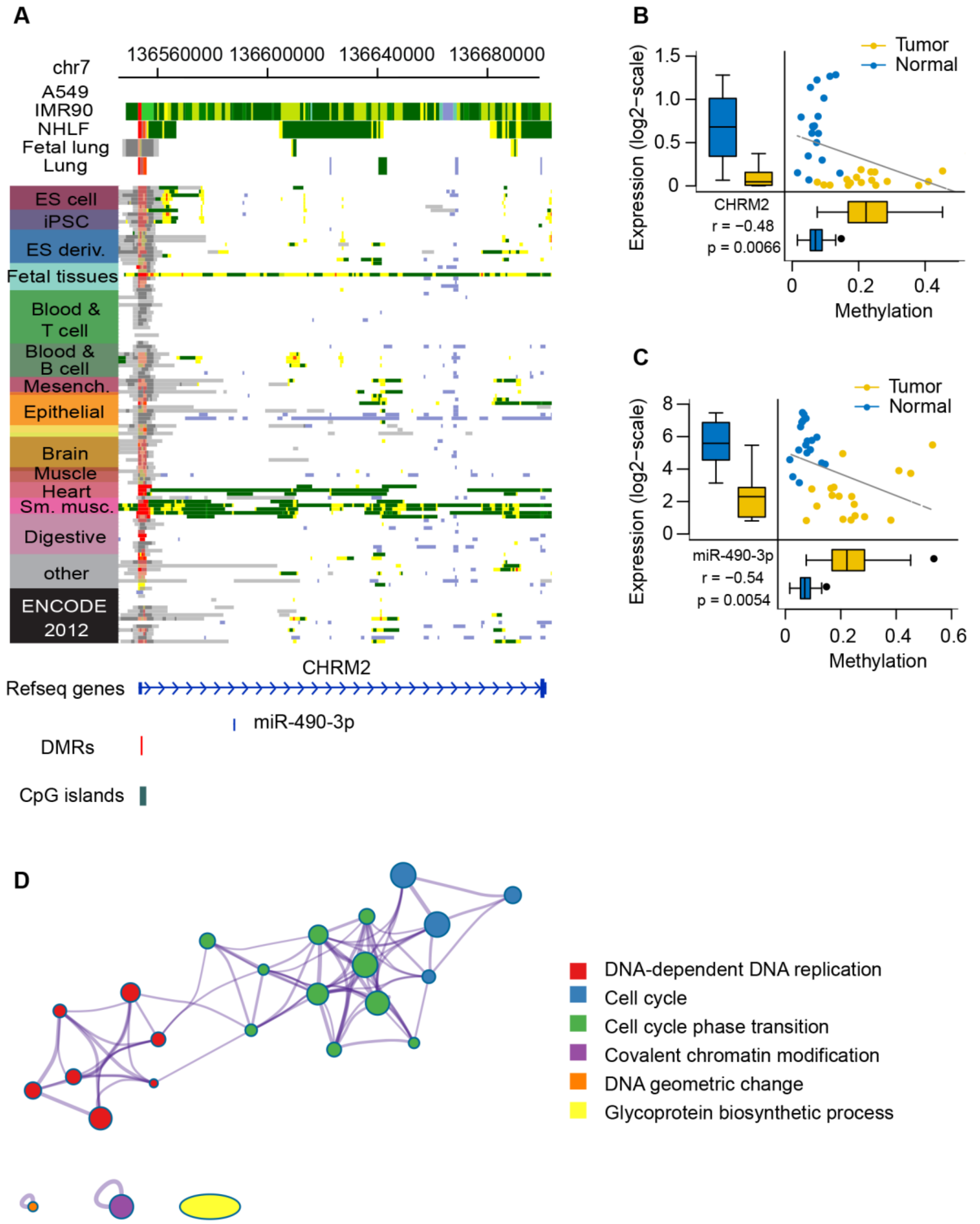

Figure S11. DNA methylation alterations control expression of miR-490-3p and its host gene CHRM2. (**A**) The epigenomic chromatin profile of DMR in miR-490-3p and CHRM2 promoter. Color-coded definitions of chromatin states are shown in Figure 2C. The position of DMR is highlighted by the red box. The CpG islands overlapped with the DMR are shown in the green box. (**B**) Scatterplots and box plots of methylation of DMR within CHRM2 promoter and its expression in the NSCLC cohort. (**C**) Scatterplots and box plots of methylation of DMR within the promoter of CHRM2 and expression of miR-490-3p in the NSCLC cohort. (**D**) Computational prediction of the biological process in which miR-490-3p is involved.

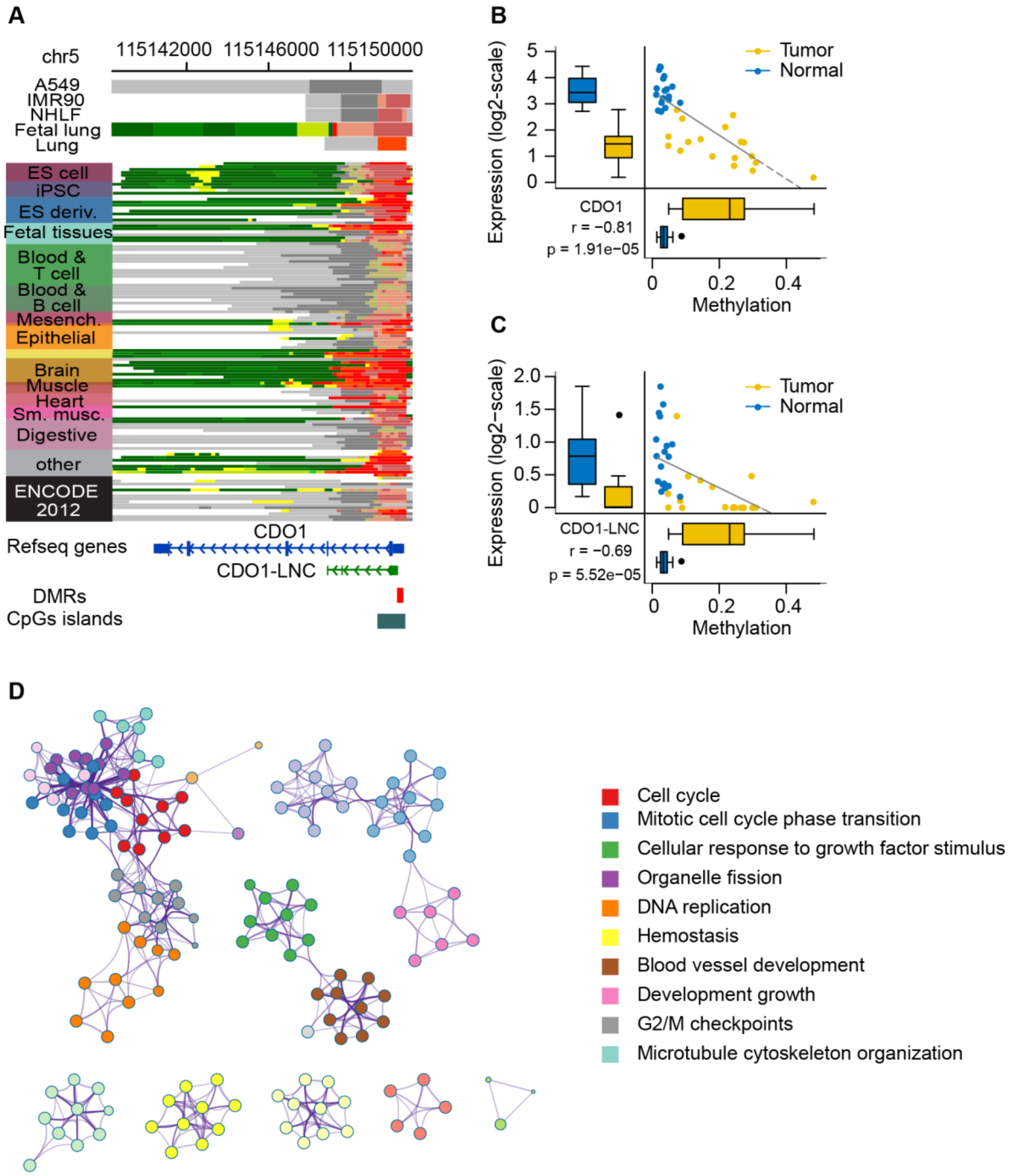

Figure S12. DNA methylation alterations control expression of CDO1-LNC and its host genes CDO1. (**A**) The epigenomic chromatin profile of DMR in CDO1-LNC and CDO1 promoter. Color-coded definitions of chromatin states are shown in Figure 2C. The position of DMR is highlighted by the red box. The CpG islands overlapped with the DMR are shown in the green box. (**B**) Scatterplots and box plots of methylation of DMR within the CDO1 promoter and its expression in the NSCLC cohort. (**C**) Scatterplots and box plots of methylation of DMR within the promoter of CDO1 and expression of CDO1-LNC in the NSCLC cohort. (**D**) Computational prediction biological process in which CDO1-LNC is involved.

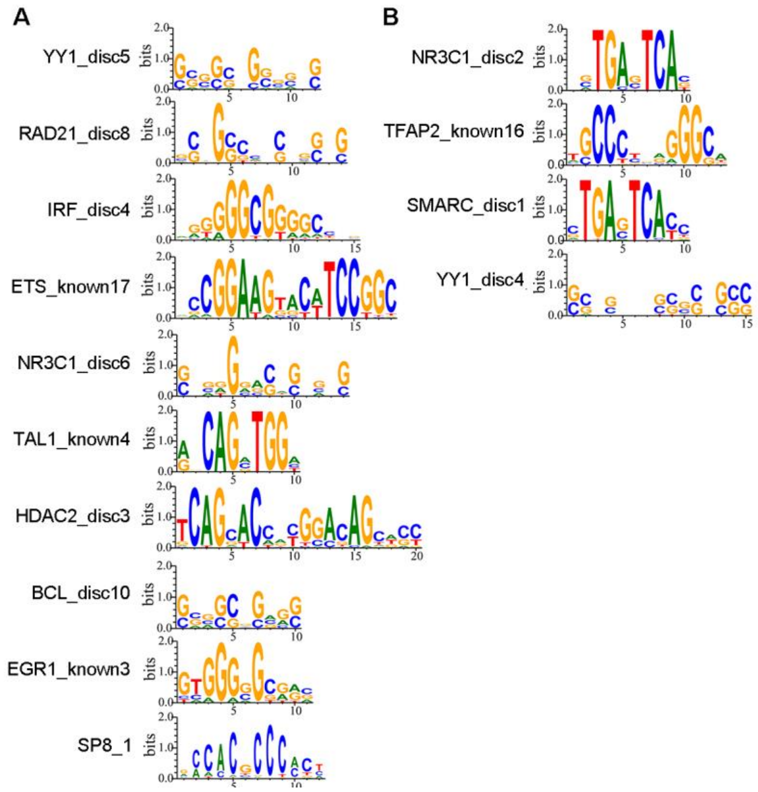

Figure S13. Display of selective transcription factor binding motifs for methylation-driven genes. (**A**) hypermethylated and down-regulated methylation-driven genes, (**B**) hypomethylated and up-regulated methylation-driven genes.

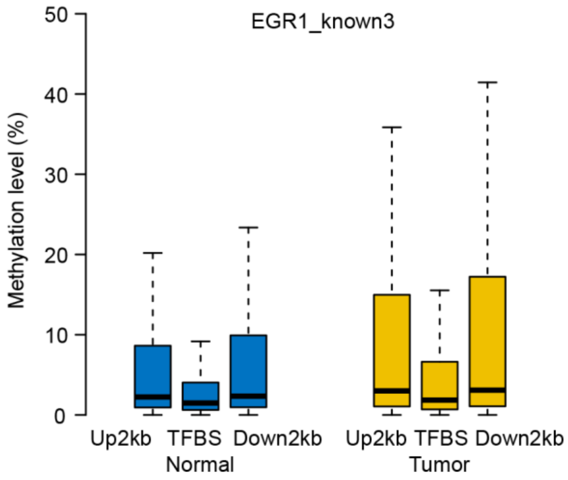

Figure S14. Distribution of methylation level for transcription factor binding motif, EGR1_known3, and ±2kb flanking the motif in tumors and adjacent normal tissues. TFBS, transcription factor binding sites.

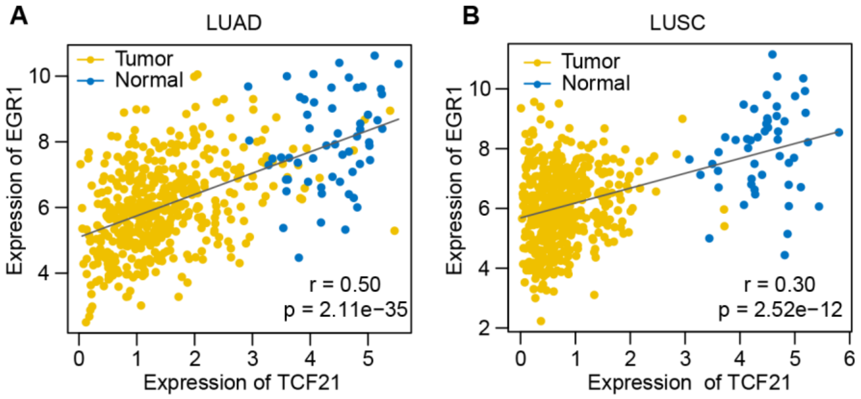

Figure S15. Correlations between expression levels of EGR1 and TCF21 in TCGA LUAD and LUSC cohorts.
